# Berberine enhances defects in the establishment of leaf polarity in *asymmetric leaves1* and *asymmetric leaves2* of *Arabidopsis thaliana*

**DOI:** 10.1007/s11103-012-9929-7

**Published:** 2012-06-10

**Authors:** Ayami Nakagawa, Hiro Takahashi, Shoko Kojima, Nobuo Sato, Kazuomi Ohga, Byung Yoon Cha, Je-Tae Woo, Kazuo Nagai, Gorou Horiguchi, Hirokazu Tsukaya, Yasunori Machida, Chiyoko Machida

**Affiliations:** 1 Plant Biology Research Center, Chubu University, 1200 Matsumoto-cho, Kasugai, Aichi 487-8501 Japan; 2 Graduate School of Bioscience and Biotechnology, Chubu University, 1200 Matsumoto-cho, Kasugai, Aichi 487-8501 Japan; 3 Research Institute for Biological Functions, Chubu University, 1200 Matsumoto-cho, Kasugai, Aichi 487-8501 Japan; 4 Department of Life Science, College of Science, Rikkyo University, 3-34-1 Nishi-Ikebukuro, Toshima-ku, Tokyo, 171-8501 Japan; 5 Graduate School of Science, The University of Tokyo, 7-3-1 Hongo, Bunkyo-ku, Tokyo, 113-0033 Japan; 6 Graduate School of Science, Nagoya University, Furo-cho, Chikusa-ku, Nagoya, Aichi 464-8602 Japan

**Keywords:** Adaxial–abaxial polarity, *asymmetric leaves2*, Berberine, Coptisine, Leaf development, Quadruplex DNA

## Abstract

Leaves develop as flat lateral organs from the indeterminate shoot apical meristem. The establishment of polarity along three-dimensional axes, proximal–distal, medial–lateral, and adaxial–abaxial axes, is crucial for the growth of normal leaves. The mutations of *ASYMMETRIC LEAVES1* (*AS1*) and *AS2* of *Arabidopsis thaliana* cause defects in repression of the indeterminate state and the establishment of axis formation in leaves. Although many mutations have been identified that enhance the adaxial–abaxial polarity defects of *as1* and *as2* mutants, the roles of the causative genes in leaf development are still unknown. In this study, we found that wild-type plants treated with berberine produced pointed leaves, which are often observed in the single mutants that enhance phenotypes of *as1* and *as2* mutants. The berberine-treated *as1* and *as2* mutants formed abaxialized filamentous leaves. Berberine, an isoquinoline alkaloid compound naturally produced in various plant sources, has a growth inhibitory effect on plants that do not produce berberine. We further showed that transcript levels of meristem-specific class 1 *KNOX* homeobox genes and abaxial determinant genes were increased in berberine-treated *as1* and *as2*. Berberine treated plants carrying double mutations of *AS2* and the large subunit ribosomal protein gene *RPL5B* showed more severe defects in polarity than did the *as2* single mutant plants. We suggest that berberine inhibits (a) factor(s) that might be required for leaf adaxial cell differentiation through a pathway independent of AS1 and AS2. Multiple pathways might play important roles in the formation of flat symmetric leaves.

**Electronic supplementary material:**

The online version of this article (doi:10.1007/s11103-012-9929-7) contains supplementary material, which is available to authorized users.

## Introduction

Leaves develop as flat lateral organs from the shoot apical meristem, and the establishment of polarity along three-dimensional axes, proximal–distal, medial–lateral and adaxial–abaxial, is crucial for the growth of normal leaves (Steeves and Sussex 1989; Hudson 2000; Tsukaya 2006; Bowman and Floyd 2008; Szakonyi et al. 2010). Thus plants appear to exploit common mechanisms that are responsible for the establishment of these axes during leaf development. Genes that are involved in the formation of each of the three axes have been identified and their functions have been analyzed (Waites et al. 1998; Bowman and Smyth 1999; Sawa et al. 1999; Byrne et al. 2000; Nemhauser et al. 2000; Eshed et al. 2001; Kerstetter et al. 2001; McConnell et al. 2001; Iwakawa et al. 2002; Pekker et al. 2005; Garcia et al. 2006). The *asymmetric leaves1* (*as1*) and *as2* mutants of *Arabidopsis thaliana* (*A. thaliana*) produce leaves abnormal in axis formation, and these mutants have pleiotropic phenotypes (Rédei and Hirono 1964; Tsukaya and Uchimiya 1997; Serrano-Cartagena et al. 1999; Byrne et al. 2000, 2001, 2002; Ori et al. 2000; Semiarti et al. 2001; Iwakawa et al. 2002, 2007; Matsumura et al. 2009; Ikezaki et al. 2010). Analyses by DNA microarray and real-time quantitative RT-PCR (qRT-PCR) revealed that a mutation of either *AS1* or *AS2* results in an increase in the transcription levels of genes for the abaxial determinants, *ETTIN*/*AUXIN RESPONSE FACTOR3* (*ETT/ARF3*), *KANADI2* (*KAN2*) and *YABBY5* (*YAB5*) as well as the class 1 *KNOX* genes, *BREVIPEDICELLUS* (*BP*), *KNAT2*, and *KNAT6* (Iwakawa et al. 2007; Takahashi et al. 2008). The class 1 *KNOX* genes (*BP*, *KNAT2*, *KNAT6*, and *SHOOT MERISTEMLESS* [*STM*]) are expressed in the shoot apical meristem and thought to function in maintaining the indeterminate cell state (Lincoln et al. 1994; Long et al. 1996; Byrne et al. 2000; Ori et al. 2000; Semiarti et al. 2001; Ikezaki et al. 2010). *AS1* encodes a myb domain protein and *AS2* encodes a plant-specific protein with an AS2/LOB domain, both of which are nuclear proteins (Byrne et al. 2000; Iwakawa et al. 2002). These proteins form a complex structure (Yang et al. 2008) (hereinafter referred to as AS1–AS2) that is involved in the establishment of proper axis formation. In addition, some mutations of genes alter leaf patterning and axis formation in the absence of either *AS1* or *AS2*: namely, ribosomal protein genes, genes involved in chromatin remodeling, histone modification, and small RNA biogenesis, and others (Ori et al. 2000; Xu et al. 2003, 2006; Kidner and Martienssen 2005; Li et al. 2005; Garcia et al. 2006; Huang et al. 2006; Yang et al. 2006; Ueno et al. 2007; Pinon et al. 2008; Yao et al. 2008; Pérez-Pérez et al. 2010; Horiguchi et al. 2011a, b; Kojima et al. 2011; Szakonyi and Byrne 2011; Ishibashi et al. 2012). Although the responsible wild-type genes also have important roles in the maintenance of the plants themselves, the roles of these wild-type genes in the establishment of leaf polarity are still unclear.

Alkaloids are nitrogen-containing low molecular weight substances that form a major class of plant-produced secondary metabolites. We screened for natural compounds that affect leaf development in *A. thaliana* and found that berberine, a benzylisoquinoline alkaloid, caused a pointed-leaf phenotype in wild-type plants. Berberine is naturally produced in various plant sources, such as the wood of *Berberis*, inner bark of *Phellodendron*, and rhizomes of *Coptis* (Grycová et al. 2007; Shitan and Yazaki 2007). The inhibitory effect of berberine on growth was reported in plant species that cannot produce berberine (Sakai et al. 2002). It has been reported that Arabidopsis plants might not produce berberine (Facchini et al. 2004); nevertheless, effects of berberine on *A. thaliana* have not been reported. For a half century, it has been known that berberine interacts with DNA and RNA (Klímek and Hnilica 1959; Yamagishi 1962; Kley and Hahn 1969; Wolfe et al. 1972). Berberine is used in biochemical research as a fluorescent probe of DNA and RNA (Hirakawa et al. 2005; Islam and Kumar 2008; Islam et al. 2009). Recently, it has been reported that berberine preferentially binds to triplex or quadruplex DNA (Lee et al. 1993; Ren and Chaires 1999; Franceschin et al. 2006; Arora et al. 2008; Ma et al. 2008; Bhadra and Kumar 2011). Guanine-rich sequences that presumably form quadruplex DNA are found throughout the genomes studied, including that of *A. thaliana* (Huppert and Balasubramanian 2005; Todd et al. 2005; Zhao et al. 2007; Mullen et al. 2010; Takahashi et al. 2012). It has been reported that sequences containing quadruplex DNA affect the transcript levels of genes located nearby (Siddiqui-Jain et al. 2002). In recent decades, berberine has been reported to possess antibacterial (Chae et al. 1999; Hu et al. 2000; Yan et al. 2008), anti-cancer (Anis et al. 2001), and antiphotooxidative activities (Kim et al. 2000). Berberine also induces apoptosis in several types of cells (Kuo et al. 1995; Hwang et al. 2006; Mantena et al. 2006) and has been used in medicines for both traditional and clinical applications. In spite of these reports and the broad uses of berberine, there have been no reports on the cause of its effects on growth inhibition and leaf morphology in plants that do not produce berberine.

In the present study, we report that wild-type Arabidopsis plants treated with berberine showed growth inhibition and changes of leaf morphology. Furthermore, we detected severe defects of leaf phenotypes related to the adaxial–abaxial polarity in *as1* and *as2* mutants treated with berberine. We discuss these effects of berberine that interfered with normal leaf development in *A. thaliana*.

## Results

### Phenotypes of wild-type plants treated with berberine

**Fig. 1:**
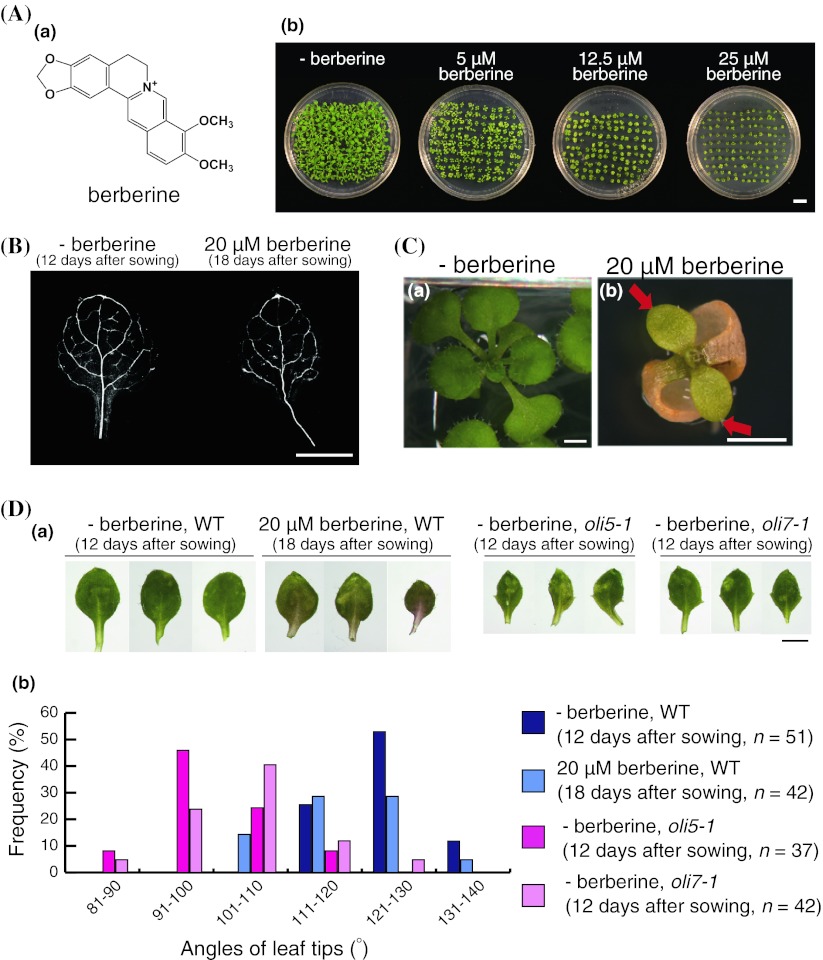
Phenotypes of Col-0 wild-type (WT) of *A. thaliana* in the presence of berberine. **A** Chemical structure of berberine (*a*) and gross morphology of Col-0 (WT) plants treated without or with 5, 12.5, or 25 μM berberine for 18 days (*b*). *Scale bar* = 1 cm. **B** Dark-field views of cleared first or second leaves of WT without (*left*) and with 20 μM berberine (*right*). *Scale bar* = 0.5 mm. **C** Typical phenotypes of WT plants grown in the absence (*a*) and the presence of 20 μM berberine (*b*). *Scale bars* = 1 mm. *Red arrows* indicate pointed leaves. **D** (*a*) First or second rosette leaves of WT, *oli5*-*1*, and *oli7*-*1* plants grown in the absence of berberine and WT plants in the presence of 20 μM berberine. *Scale bars* = 1 mm. (*b*) A histogram of tip angles of the first or second leaves of WT, *oli5*-*1*, and *oli7*-*1* plants grown in the absence of berberine and of WT plants in the presence of 20 μM berberine

When we treated plants with berberine (Fig. 1Aa), they displayed obviously severe effects of growth inhibition with decreased leaf size (Fig. 1Ab), similar to those observed in plants treated with trichostatin A (Ueno et al. 2007). As shown in Fig. 1B, the sizes of the first and second rosette leaves in plants at 18 days after sowing with 20 μM berberine were comparable to those at 12 days after sowing without berberine. Furthermore, the venation pattern of berberine-treated leaves was simpler than that of untreated leaves (Fig. 1B). We also observed pointed leaves at 20 and 25 μM concentrations of berberine similar to those observed in *oligocellula5*-*1* (*oli5*-*1*) and *oligocellula7*-*1* (*oli7*-*1*) mutant plants, each of which has a mutation in the large subunit of ribosomal protein gene *RPL5A* and *RPL5B*, respectively (Horiguchi et al. 2006; Fujikura et al. 2009; Fig. 1Cb, D; Table 1). These *oli5*-*1* and *oli7*-*1* mutants were identified as showing decreased palisade leaf cell numbers and pointed-leaf phenotypes. The leaf polarity defects of *oli5*-*1* were rather more severe than those of *oli7*-*1* (Horiguchi et al. 2011a). We quantified the pointed-leaf phenotype by measuring the tip angles of the first and second leaves. For wild-type plants grown without berberine, the mean tip angle was 123.7°, whereas those for berberine-treated wild-type and the *oli5*-*1* and *oli7*-*1* mutant plants were 118.7°, 98.6°, and 104.1°, respectively (*p* < 0.01). In this paper, we categorized leaves with tip angles less than 110° as ‘pointed leaves’. As shown in Fig. 1Db, there were no leaves with tip angles less than 110° seen in wild-type plants without berberine; whereas, 14 % of wild-type leaves treated with berberine (6 out of 42 leaves), 92 % of *oli5*-*1* leaves (34 out of 37 leaves), and 69 % *oli7*-*1* leaves (34 out of 42 leaves) had leaf-tip angles smaller than 110°.

**Table 1:** Frequencies of plants with abnormal leaves and lethal phenotypes after growth on medium that contained berberine

	Number of plants with pointed leaves				Number of plants with trumpet-like and filamentous leaves				Number of plants without true leaves			
Berberine (μM)	0	12.5	20	25	0	12.5	20	25	0	12.5	20	25
Col-0 (WT)	0 ± 0	7 ± 9	8 ± 8	7 ± 7	0 ± 0	2 ± 1	3 ± 2	5 ± 6	0 ± 0	3 ± 2	11 ± 6	17 ± 14
*as1*-*1*	0 ± 0	8 ± 6	8 ± 5	9 ± 6	0 ± 0	3 ± 3	13 ± 4	18 ± 10	0 ± 0	1 ± 1	20 ± 21	27 ± 23
*as2*-*1*	0 ± 0	5 ± 1	6 ± 2	3 ± 3	0 ± 0	8 ± 7	35 ± 16	28 ± 16	0 ± 0	2 ± 3	14 ± 12	34 ± 21

Plants (*n* = 96) were used for each berberine treatment. Each value indicates the mean and the standard deviation of 4 batches of experiments

The trumpet-like or filamentous leaves on each plant were counted under the stereomicroscope at 18 days after sowing

### Phenotypes of *as1*-*1* and *as2*-*1* plants treated with berberine

**Fig. 2:**
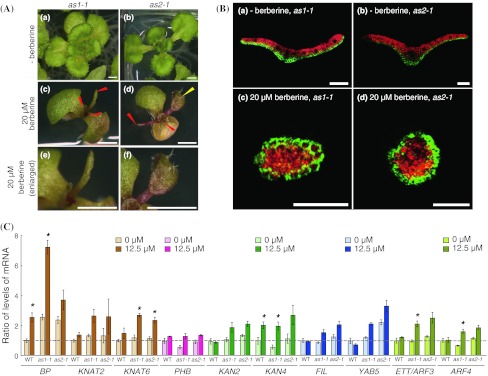
Phenotypes of *as1*-*1* and *as2*-*1* mutants of *A. thaliana* in the presence of berberine. **A** Typical phenotypes of *as1*-*1* and *as2*-*1* plants grown in the absence (*a*, *b*) and the presence of 20 μM berberine (*c*, *d*, *e*, *f*). (*a*, *c*, *e*) *as1*-*1* plants, (*b*, *d*, *f*) *as2*-*1* plants. (*e*, *f*) Enlarged filamentous (*e*) and trumpet-like (*f*) leaves are seen in panels (*c*) and (*d*), respectively. *Red arrowheads* on *as1*-*1* and *as2*-*1* plants show filamentous leaves. A *yellow arrowhead* indicates a trumpet-like leaf. *Scale bars* = 1 mm in (*a*) to (*d*), 0.5 mm in (*e*) and (*f*). **B** *as1*-*1* *FILp:GFP* (*a*, *c*), and *as2*-*1* *FILp:GFP* plants (*b*, *d*) were grown on medium without and with 20 μM berberine for 18 days. Transverse sections of leaves are shown. *Green signals* from GFP; *red*, autofluorescence. *Scale bars* = 100 μm. **C** Changes in transcription levels of genes involved in the maintenance of shoot apical meristem and leaf adaxial–abaxial polarity in *A. thaliana*. RNA was extracted from 14-day-old shoots of WT, *as1*-*1*, or *as2*-*1* plants. Each value was normalized by reference to the level of *TUA3* transcripts. *Error bars* show standard deviations (*n* = 3). Values indicated by *dashed lines* are shown relative to the values for WT plants treated with 0 μM berberine. An *asterisk* indicates over a twofold significant increase from each plant not treated with berberine (*p* < 0.01 by Student’s *T* test)

Mutations that cause pointed leaves, such as *oli5*-*1* and *oli7*-*1*, have been previously reported to enhance the phenotypes of *as1* and *as2* mutants (Fig. 1Da; Pinon et al. 2008; Yao et al. 2008; Horiguchi et al. 2011a; Szakonyi and Byrne 2011; Ishibashi et al. 2012). Therefore, we examined the phenotypes of wild-type and *as1*-*1* and *as2*-*1* mutant plants treated without and with 12.5, 20, and 25 μM berberine. The results are given in Table 1. The pointed-leaf phenotype was found in 7, 8, and 7 % of wild-type plants as well as in *as1*-*1* and *as2*-*1* plants treated with 12.5, 20, and 25 μM berberine, respectively (Table 1). Wild-type plants seldom showed radialized leaves (Table 1), whereas 3, 13, and 18 % of *as1*-*1* and 8, 35, and 28 % of *as2*-*1* plants had trumpet-like leaves and filamentous leaves indicative of a defect in adaxial–abaxial polarity at 12.5, 20, and 25 μM berberine, respectively (Fig. 2Ac, Ad, Ae, Af; Table 1). We also observed plants without true leaves. When grown with 25 μM berberine, true leaves were not seen on 17 % of wild type, 27 % of *as1*-*1*, and 34 % of *as2*-*1* plants (Table 1). These results indicated that *as1*-*1* and *as2*-*1* mutant plants were more sensitive than the wild-type plants to berberine treatment.

Since trumpet-like and filamentous leaves are often generated in plants having defective adaxial–abaxial polarity determination, we examined whether these leaf phenotypes are associated with transcript levels of certain genes in the presence of berberine treatment. To examine the effect of berberine treatment on leaf adaxial–abaxial polarity, we used green fluorescent protein (GFP) under the control of the *FILAMENTOUS FLOWER* (*FIL*) promoter (*FILp:GFP*), which is expressed in abaxial cells of leaf primordia (Watanabe and Okada 2003). Strong signals were detected in cells located at peripheral positions in filamentous leaves in the presence of 20 μM berberine (Fig. 2Bc, Bd), whereas no signals were detected in cells located on the adaxial side of leaves of *as1*-*1* and *as2*-*1* plants in the absence of berberine (Fig. 2Ba, Bb).

We next examined the accumulated transcript levels of genes involved in leaf adaxial–abaxial polarity and class 1 *KNOX* family genes in 14-day-old shoots treated with 12.5 μM berberine. Figure 2C shows the changes in the accumulated transcript levels of representative genes. Supplementary Table S2 shows relative changes in transcript levels of all the genes analyzed in each plant. When the wild-type plants were treated with 12.5 μM berberine, the accumulated transcript levels of *BP* and *KAN4* showed significant increases over twofold higher than those levels of the non-treated wild type (Fig. 2C). Accumulated transcript levels of *BP*, *KAN4*, *ETT/ARF3*, and *ARF4* in either the *as1*-*1* or *as2*-*1* or both plants treated with berberine at 12.5 μM showed significant increases over twofold higher than those of non-treated *as1*-*1* or *as2*-*1* plants (Fig. 2C; Supplementary Table S2). In contrast, the accumulated transcript levels of the adaxial determinant *HD*-*ZIPIII* genes *PHABULOSA* (*PHB*), *PHAVOLUTA* (*PHV*), and *REVOLUTA* (*REV*) were not significantly changed by berberine treatment in wild-type, *as1*-*1*, and *as2*-*1* plants (Supplementary Table S2).

### Phenotypes of plants treated with other isoquinoline alkaloid compounds

**Fig. 3:**
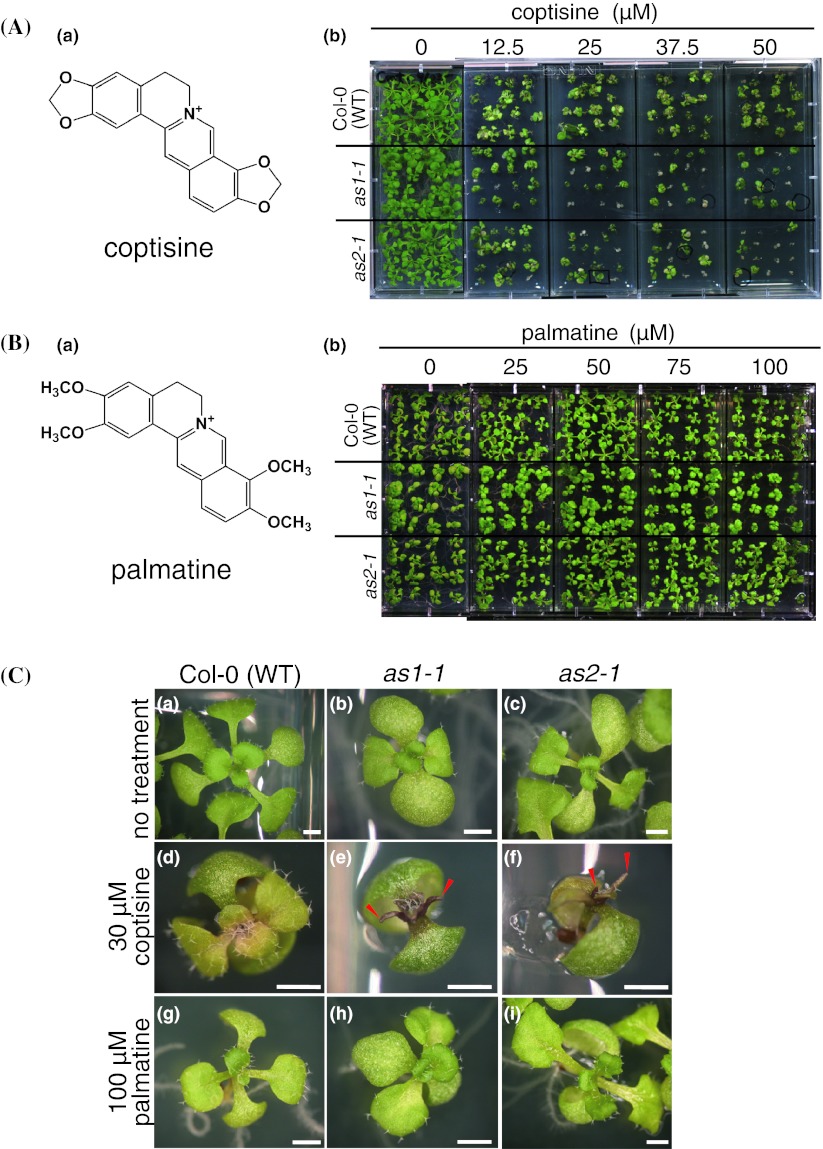
Phenotypes of Col-0 (WT), *as1*-*1*, and *as2*-*1* mutants of *A. thaliana* in the presence of the isoquinoline alkaloids coptisine and palmatine. **A** Chemical structure of coptisine (*a*) and gross morphology of WT, *as1*-*1*, and *as2*-*1* plants grown on MS medium without and with coptisine for 21 days (*b*). **B** Chemical structure of palmatine (*a*) and gross morphology of WT, *as1*-*1*, and *as2*-*1* plants grown on MS medium without and with palmatine for 18 days (*b*). **C** Leaf phenotypes of plants grown without (*a*, *b*, *c*) and with 30 μM coptisine (*d*, *e*, *f*) and 100 μM palmatine (*g*, *h*, *i*) for 18 days. (*a*, *d*, *g*) WT plants, (*b*, *e*, *h*) *as1*-*1* plants, (*c*, *f*, *i*) *as2*-*1* plants. *Red arrowheads* in panels (*e*, *f*) show filamentous leaves. *Scale bars* = 1 mm

To understand which structure of the isoquinoline alkaloid compounds is responsible for their function, we next examined whether two other isoquinoline alkaloid compounds, coptisine and palmatine, having chemical structures similar to that of berberine (Fig. 3Aa, Ba), affect the phenotypes of *as1* and *as2* leaves. The sensitivity of plants to coptisine was similar to that of berberine (Fig. 3Ab). Trumpet-like and filamentous leaves were observed at concentrations from 20 to 50 μM in *as1*-*1* and *as2*-*1* plants (Fig. 3Ce, 3Cf), whereas these abnormal leaves were not observed in wild-type plants (Fig. 3Cd; Table 2). Trumpet-like and filamentous leaves were observed in 28 % (27 out of 96 plants) and 43 % (42 out of 96 plants) of *as1*-*1* and *as2*-*1* plants at 40 μM coptisine, respectively (Table 2). Plants without true leaves were not observed with coptisine treatment, although growth was inhibited. In contrast, treatments with 100 μM palmatine had no effect on either growth or leaf morphology (Fig. 3Bb, Cg, Ch, Ci). These results suggested that some specific conformation or residues might be important for affecting plant growth and morphology.

**Table 2:** Frequencies of plants with abnormal leaves and lethal phenotypes after growth on medium that contained coptisine

	Number of plants with pointed leaves						Number of plants with trumpet-like and filamentous leaves						Number of plants without true leaves					
Coptisine (μM)	0	20	25	30	40	50	0	20	25	30	40	50	0	20	25	30	40	50
Col-0 (WT)	0	0	0	0	0	0	0	0	0	0	0	1	0	0	0	0	3	2
*as1*-*1*	0	1	0	1	1	1	0	8	16	21	27	21	0	1	0	0	7	29
*as2*-*1*	0	2	0	3	1	0	0	16	37	42	42	37	0	1	1	3	11	30

Plants (*n* = 96) were used for each coptisine treatment

The filamentous or trumpet-like leaves on each plant were counted under the stereomicroscope at 18 days after sowing

### Phenotypes of plants treated with a DNA intercalator

**Fig. 4:**
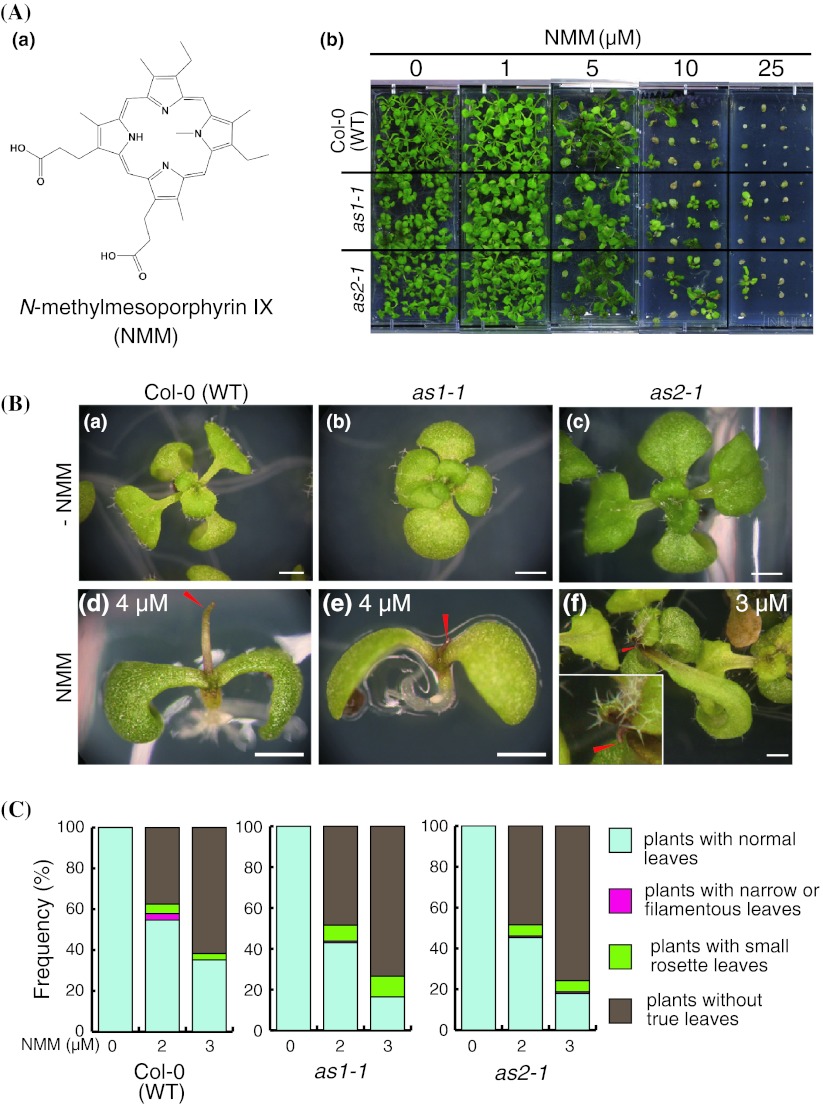
**A** Chemical structure of NMM (*a*) and gross morphology of Col-0 (WT), *as1*-*1*, and *as2*-*1* plants grown on MS medium without and with NMM for 21 days (*b*). **B** Leaf phenotypes of plants grown in the absence (*a*, *b*, *c*) and the presence of 3 or 4 μM NMM (*d*, *e*, *f*) for 14 days (except panel f shows 19 days after sowing *as2*-*1* plant). (*a*, *d*) WT plants, (*b*, *e*) *as1*-*1* plants, (*c*, *f*) *as2*-*1* plants. The inset in panel (*f*) shows enlarged filamentous leaves of an *as2*-*1* plant. *Red arrowheads* in panels (*d*, *e*, *f*) show filamentous leaves. *Scale bars* = 1 mm. **C** Frequencies of plants with abnormal leaves and lethal phenotypes after growth on medium that contained G-quadruplex ligand NMM. Plants (*n* = 128) were used for each NMM treatment. The growth and leaf phenotypes on each plant were scored under the stereomicroscope

Since it has been reported that berberine forms complexes with duplex, triplex, and quadruplex DNA (Klímek and Hnilica 1959; Yamagishi 1962; Kley and Hahn 1969; Lee et al. 1993; Ren and Chaires 1999; Franceschin et al. 2006), we attempted to treat *as1* and *as2* mutant plants with one of the DNA intercalators, *N*-methylmesoporphyrin IX (NMM), which has been known as a major quadruplex DNA ligand (Arthanari et al. 1998). NMM treatment severely inhibited growth of plants at 10 μM and plants rarely germinated at 25 μM (Fig. 4Ab). NMM treatment strongly inhibited true leaf formation. As shown in Fig. 4C, the sensitivity to NMM was stronger in *as1*-*1* and *as2*-*1* plants than in wild-type plants: 73 % of *as1*-*1* plants and 75 % of *as2*-*1* plants were deficient in true leaf formation, whereas 61 % of the wild-type plants were deficient at 3 μM NMM. Plants treated with NMM showed filamentous leaves in all wild-type, *as1*-*1*, and *as2*-*1* plants at low concentrations of NMM; although we did not detect a significant difference in the frequencies of filamentous leaf formation between wild-type and the *as1*-*1* or *as2*-*1* mutant plants (Fig. 4Bd, Be, Bf).

### Increased sensitivity of berberine by a mutation in cytosolic ribosomal protein *RPL5B*

**Fig. 5:**
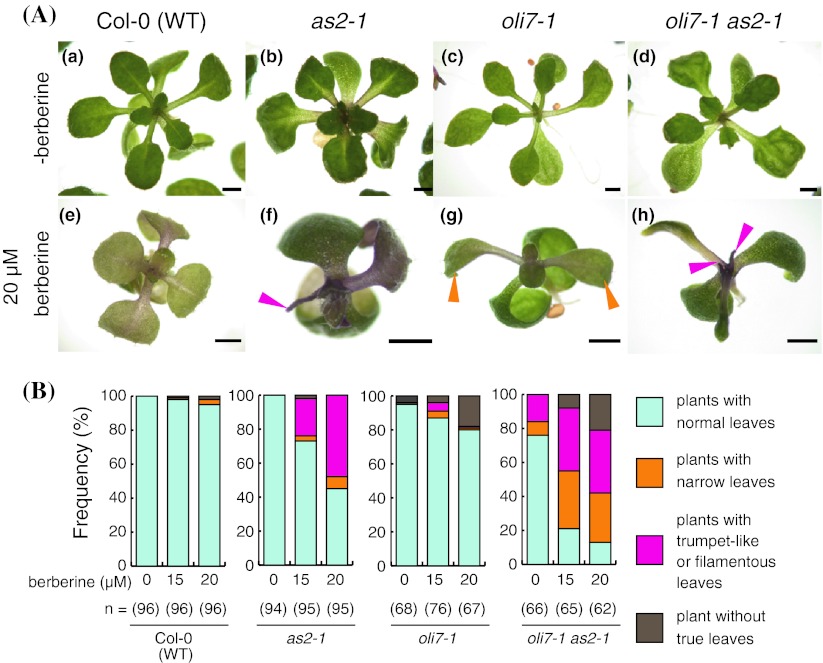
**A** Leaf phenotypes of plants grown without (*a*, *b*, *c*, *d*) and with 20 μM berberine (*e*, *f*, *g*, *h*) for 18 days. (*a*, *e*) WT plants, (*b*, *f*) *as2*-*1* plants, (*c*, *g*) *oli7*-*1* plants, (*d*, *h*) *oli7*-*1 as2*-*1* plants. *Red and orange arrowheads* show filamentous leaves and fairly narrow leaves, respectively. *Scale bars* = 1 mm. **B** Frequencies of plants with normal leaves, abnormal leaves, and without true leaves after growth on medium that contained 0, 15, or 20 μM berberine. Number of plants used for each treatment is shown below the bar graph. The growth and leaf phenotypes of each plant were scored under the stereomicroscope

Most DNA intercalators are also known to bind to ribosomal RNA, a component of the ribosome complex, and result in ribosome degradation (Wolfe et al. 1972). We treated wild-type, *oli7*-*1*, and *oli7*-*1* *as2*-*1* double mutant plants with berberine. Berberine treatment severely inhibited the growth of *oli7*-*1* plants at 15 and 20 μM (Fig. 5A). When we treated these plants with berberine, 4 and 18 % of *oli7*-*1* plants did not have true leaves at 15 and 20 μM berberine, respectively, whereas very lower frequencies (1 and 2 %) of wild-type plants appeared without true leaves at 15 and 20 μM berberine (Fig. 5B). The *oli7*-*1* plants treated with 20 μM berberine also displayed the narrower leaf phenotype (orange arrowheads in Fig. 5Ag). When treated with 15 and 20 μM berberine, the narrow-leaf phenotype was found in 4 and 1 % of *oli7*-*1* plants, 34 and 29 % of *oli7*-*1* *as2*-*1* double mutant plants, and 1 and 3 % of wild-type plants, respectively (Fig. 5A, B). Trumpet-like and filamentous leaves were also observed at 15 and 20 μM berberine treatment in 5 and 1 % of *oli7*-*1* plants, respectively, which are comparable frequencies to those of wild-type plants. As reported previously (Horiguchi et al. 2011a), 17 % of the *oli7*-*1* *as2*-*1* double mutants displayed a filamentous-leaves phenotype in the absence of berberine, and the frequencies of the filamentous-leaves phenotype were approximately twofold increased (37 %) in the presence of both 15 and 20 μM berberine.

### The effects of berberine on transcript levels in wild-type plants

To know how berberine affects leaf cells in wild-type plants, we used Affymetrix ATH1 microarrays to analyze transcript levels of genes in Arabidopsis plants grown on MS medium with and without 12.5 μM berberine for 14 days. Microarray analysis showed that transcript levels of 3,854 out of 22,591 genes on the ATH1 gene chip (excluding the mitochondrial genome) were changed by berberine treatment (Supplementary Table S3). To date, mutations of many ribosomal protein genes and other genes have been identified that enhance *as1* and or *as2* leaf phenotypes (Ori et al. 2000; Xu et al. 2003, 2006; Kidner and Martienssen 2005; Li et al. 2005; Garcia et al. 2006; Huang et al. 2006; Yang et al. 2006; Ueno et al. 2007; Pinon et al. 2008; Yao et al. 2008; Pérez-Pérez et al. 2010; Horiguchi et al. 2011a, b; Kojima et al. 2011; Szakonyi and Byrne 2011; Ishibashi et al. 2012). We analyzed whether the expression of these known enhancer genes was repressed by berberine treatment; however, transcript levels of such genes were not changed significantly by berberine.

## Discussion

In the present study, we found that berberine affected the establishment of leaf polarity in the *as1* or *as2* mutant plants of *A. thaliana*. Our results by treatments with low molecular weight compounds implicated the existence of a novel factor(s), which is involved together with *AS1* and *AS2* in the establishment of adaxial cells of leaves.

### Berberine affects leaf development of Arabidopsis and upregulates the transcript levels of class 1 *KNOX* and leaf abaxial determinant genes

**Fig. 6:**
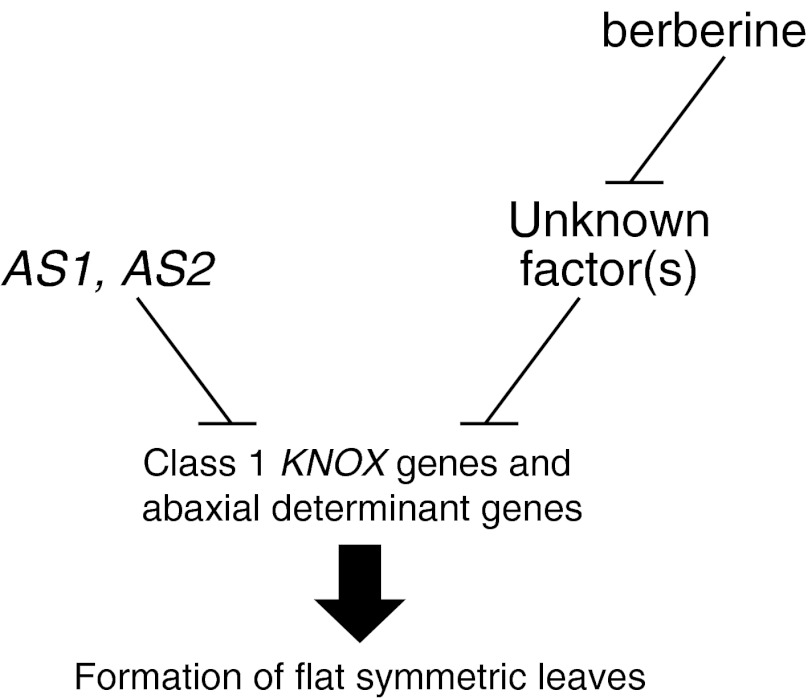
Suggested roles of *AS1*, *AS2*, and berberine in *A. thaliana* leaf development. Berberine may inhibit an unknown factor(s) that might be involved in the formation of flat symmetric leaves together with *AS1* and *AS2*

When we treated the *as1*-*1* and *as2*-*1* mutant plants with berberine, they produced abaxialized filamentous leaves (Fig. 2A). Berberine might inhibit a function(s) of a novel factor(s) that is involved in adaxialization independently from AS1–AS2 during leaf development. Namely, the factor(s) and AS1–AS2 might share the same target to control the adaxialization of leaves through independent pathways (Fig. 6). It has been reported that the AS1–AS2 complex directly represses the transcription of *BP* and *KNAT2* genes (Guo et al. 2008). Transcription of the abaxial determinant genes *ETT/ARF3*, *KAN2*, and *YAB5* was also repressed by *AS1* and *AS2* (Iwakawa et al. 2007). Our real-time qRT-PCR results also showed that berberine treatment increased transcript levels of *BP* and *KAN4* genes in wild-type plants, and the class 1 *KNOX* genes *BP* and *KNAT6* and the abaxial determinant genes *KAN4*, *ETT/ARF3*, and *ARF4* in the *as1* and *as2* mutants. These results suggest that the novel factor(s) inhibited by berberine somehow represses *BP* and *KAN4* genes through an unidentified pathway that would act in parallel with AS1–AS2 for the establishment of leaf polarity, resulting in the formation of flat symmetric leaves in *A. thaliana* (Fig. 6).

### Factors inhibited by berberine and coptisine that are required for the establishment of leaf polarity in parallel with *AS1* and *AS2*

Berberine, coptisine, and palmatine are close structural analogs of each other. They bear the same tetracyclic structure (7,8,13,13a-tetrahydroberbinium), but differ in the nature of the substituents at positions 2 and 3 on the benzo ring, being methylene dioxy for berberine and coptisine, and dimethoxy for palmatine. Similarly to berberine, coptisine and palmatine also have a buckled structure. Recently, berberine and coptisine have been reported to inhibit the receptor activator of nuclear factor-kappaB ligand (RANKL)-induced osteoclast differentiation from osteoclast precursors, the survival of mature osteoclasts, and ovariectomy-stimulated bone resorption in rats (Li et al. 1999; Hu et al. 2008; Lee et al. 2008; Lee et al. 2012). In contrast, the inhibitory effect of palmatine on osteoclast differentiation and function is markedly weaker than that of berberine (Hu et al. 2008; Lee et al. 2010, 2012). Since both berberine and coptisine affected leaf development, the methylene dioxy substituent at positions 2 and 3 on the benzo ring might be responsible for determining their targets, although their targets might be different from that in RANKL-induced osteoclast differentiation in rats.

### Multiple pathways are involved in the adaxial development of *as1* and *as2* leaves

Our microarray analysis showed that berberine negatively affected the expression of many genes; however, we did not detect decreased transcript levels of those genes that have been known to interact genetically with *AS1* and/or *AS2*. To seek for the means by which berberine repressed such unknown factor(s), we considered several possible mechanisms of repression (inhibition), such as via berberine intercalation with secondary structures of DNA (quadruplex and triplex) and RNA, or by binding with proteins (Meyerson et al. 1978). Berberine treated plants having double mutations of *AS2* and *RPL5B* showed more severe defects in polarity than did those *as2* single mutant plants (Fig. 5A, B). These results suggest that berberine might repress other ribosomal components in the same pathway with RPL5B/OLI7/ANG3. Our genetic analysis also suggests that a process mediated by an unknown factor(s), whose function is inhibited by berberine, is the distinct process involving *RPL5B* for the establishment of leaf polarity. As we previously suggested in Kojima et al. (2011), the adaxial development of leaves might be controlled independently by multiple pathways that include the products of *ELO3*, *RDR6*-*AGO7*, and *HDT1/HDT2*. Considering that plants treated with another G-quadruplex ligand NMM also showed filamentous leaves (Fig. 4B), it might also be possible that berberine represses unknown factors via intercalating secondary DNA/RNA structures such as G-quadruplex DNAs. Especially, a DNA sequence that putatively forms a G-quadruplex is found in the transcription initiation site of ribosomal DNA in both the human (Hanakahi et al. 1999) and Arabidopsis genomes (Takahashi et al. 2012). It remains to be analyzed whether berberine binds to the rDNA region or to rRNA with the resulting impaired formation of leaf polarity. Taken together, the adaxial development of leaves is likely to be controlled independently by multiple pathways on the *as1* or *as2* background.

Since adaxial defects of leaves caused by berberine are not obvious in wild-type plants (Fig. 2C), the wild-type AS1–AS2 function can replace the polarity establishment function of an unknown factor(s) that is inhibited by berberine. That is to say, the wild-type *AS1* and *AS2* genes appear to attenuate deficiencies resulting from treatments with berberine and coptisine. Further investigations of the molecular functions of AS1 and AS2 proteins will allow us to understand how they could regulate gene expression by the presently unknown mechanism(s).

Our experiments are representative of a new method to identify novel factors that take part in leaf development by using the *as1* or *as2* mutant. The combination of comprehensive chemical screening and genetic analysis may make it possible to elucidate new events involved in the establishment of leaf polarity.

## Materials and methods

### Plant strains and growth conditions

*Arabidopsis thaliana* ecotype Col-0 (CS1092) and the mutants *as1*-*1* (CS3374) and *as2*-*1* (CS3117) were obtained from the Arabidopsis Biological Resource Center (ABRC) (Columbus, OH, USA). We outcrossed *as2*-*1* with Col-0 three times and *as1*-*1* with Col-0 once and used the progeny for our experiments (Semiarti et al. 2001). The *oli* mutants, back-crossed to Col-0 more than three times, were used for our experiments (Horiguchi et al. 2006; Fujikura et al. 2009). For the analysis of plants, seeds were surface-sterilized with 0.25 % sodium hypochlorite/0.1 % Triton X-100 solution and sown on Murashige and Skoog (MS) medium (Wako Pure Chemicals, Osaka, Japan) supplemented with 2 % (w/v) sucrose and 0.8 % agar. After 2 days at 4 °C in darkness, plants were transferred to a daily regimen of 8 h of darkness and 16 h of white light at 50 μmol m^−2^ S^−1^ at 22 °C, as described previously (Semiarti et al. 2001). Berberine chloride and palmatine chloride were purchased from Sigma-Aldrich (St. Louis, Missouri, USA). Coptisine chloride was purchased from Wako Pure Chemicals. NMM was purchased from Frontier Scientific, Inc. (Logan, Utah, USA). For chemical treatments, berberine, coptisine, or palmatine dissolved in dimethyl sulfoxide, or NMM dissolved in H_2_O was mixed with MS agar medium at 1–100 μM concentrations and immediately dispensed into plastic multi-dishes (multidish 267061, Thermo Fisher Scientific K. K., Yokohama, Japan). Ages of plants are given in terms of the number of days after sowing.

### Morphological observation of leaves

Whole leaves were observed under a stereoscopic microscope (Stemi SV11, Carl Zeiss Inc., Oberkochen, Germany). Leaves were fixed in a formalin-acetic acid-alcohol solution and cleared in a chloral hydrate solution as previously described (Tsuge et al. 1996).

### Quantification of angles of leaf tips

First or second true leaves were dissected from seedlings treated with and without berberine, mounted on a glass slide, and photographed under a stereoscopic microscope (Stemi SV11, Carl Zeiss). We drew a line along the mid vein from the one-eighth distal area of a leaf blade and a vertical line on leaves of the digital image using Adobe Illustrator software (Adobe Systems Incorporated, San Jose, CA, USA). We then drew lines toward the leaf tips from the crossing points with the vertical line and the outline of the leaf blade. The resulting angles were calculated by using the software ImageJ (http://rsbweb.nih.gov/ij/). Statistical significance was calculated by Student’s *T* test and *F* test by using Excel (Microsoft Corporation, Tokyo, Japan).

### Histological analyses

Thin sections and cleared specimens were prepared as described previously (Semiarti et al. 2001). Leaf sections were cut by hand, and GFP fluorescence images were obtained with a confocal laser microscope (LSM510, Carl Zeiss Inc., Oberkochen, Germany) according to Ueno et al. (2007).

### Real time quantitative reverse transcriptase-polymerase chain reaction (qRT-PCR)

Shoots were harvested 14 days after seeds were sown and immediately frozen in liquid nitrogen and stored at −80 °C. Total RNA was isolated from 14-day-old seedlings with the RNeasy Plant Mini Kit (Qiagen, Valencia, CA) according to the manufacturer’s instructions. Sample volumes were normalized as described by Iwakawa et al. (2007). The primer pairs are shown in Supplementary Table S1. For the analysis of RNA levels in Arabidopsis by real-time qRT-PCR, we prepared 10 μg of total RNA. Reverse transcription was carried out with ReverTra Ace (TOYOBO, Osaka, Japan). PCR was performed in the presence of the double-stranded DNA-specific dye SYBR Green (Applied Biosystems, Lincoln, CA). Amplification was monitored in real time with the Applied Biosystems StepOnePlus Real-Time PCR system (Applied Biosystems) according to the supplier’s recommendations. The mean value of three technical replicates was normalized by using the *TUBULIN α*-*3* (*TUA3*) transcript as a control. Statistical significance was calculated by Student’s *T* test and *F* test by using Excel.

### Microarray

Seedlings of wild-type plants grown with and without 12.5 μM berberine for 14 days were harvested and total RNA was extracted as described in the ‘real time qRT-PCR’ section, above. For microarray analysis, the quality and purity of the RNA were confirmed with an Ultrospec 2100 pro (GE Healthcare UK Ltd, Amersham Place, England). Total RNA samples (8 μg) were reverse-transcribed, yielding double-stranded cDNA, which was transcribed in vitro in the presence of biotin-labeled nucleotides with an IVT Labeling Kit (Affymetrix Inc., Santa Clara, CA), and purified. Labeled cRNA was fragmented and hybridized to Affymetrix ATH1 GeneChip arrays for 16 h at 45 °C according to Affymetrix protocols. Arrays were washed on an Affymetrix Fluidics Station 450 and measured for fluorescence intensity with an Affymetrix GeneChip Scanner 3000. The raw data were processed by using Affymetrix Gene Chip Operating Software (GCOS; Version 1.4.0.036).

### Microarray analysis

We calculated the expression signals for two samples and the log2 ratio for berberine-treated samples against the control sample by GCOS. The detection calls and change calls were calculated based on the signal-intensity ratio between perfect-match and miss-match oligos on the array. In this experiment, we excluded those genes (6,782 genes) for which two samples showed an absent or marginal call (i.e., detection call determined by GCOS, based on the *p* value of the one-sided Wilcoxon signed-rank test: absent call means *p* ≥ 0.065 and marginal call means 0.065 > *p* ≥ 0.05), because absent indicates that the expression signal was undetectable. We also excluded those genes (18,737 genes) for which two samples showed no change calls (i.e., change call determined by GCOS: no change call means (1–0.006) ≥ *p* ≥ 0.006 for the one-sided Wilcoxon signed-rank test), because no change indicates that the expression signal is almost equal to that of the control sample.

## Electronic supplementary material

Below is the link to the electronic supplementary material.

Supplementary material 1 (PDF 693 kb)
