# Supplementary material for: Berberine enhances defects in the establishment of leaf polarity in asymmetric leaves1 and asymmetric leaves2 of Arabidopsis thaliana
Source: Plant Mol Biol. 2012 Jun 10;79(6):569–81. doi: 10.1007/s11103-012-9929-7 (PMC3402677; doi:10.1007/s11103-012-9929-7)
Supplement: Supplementary file 1 — Supplementary material 1 (PDF 693 kb) [file 11103_2012_9929_MOESM1_ESM.pdf]

Supplementary Table S1. Primers used in this study.

primer name	sequence
BP-F	5'- TGTTGTTTCCACATATGAGCTCTCT -3'
BP-R	5'- TCATGATCAGATCGGAAGCAAT -3'
KNAT2-F	5'- TTCCGCTCGACGGAAGAC -3'
KNAT2-R	5'- AATCGGACGGCATCATCAAC -3'
KNAT6-F	5'- GATGTCACCGGAGAGTCTCATG -3'
KNAT6-R	5'- CGGCGGAGGAACATAGCA -3'
STM-F	5'- CTCCTCCCCAAGGAACTAAGAAC -3'
STM-R	5'- TCCTCCTGCAACGATTTCG -3'
KAN1-F	5'- CCACGCGCGGTTTGTT -3'
KAN1-R	5'- CGACTTTGGAGTTGCTCTTTCA -3'
KAN2-F	5'- AAGGAACTAGATGGAAAGTGCTCAA -3'
KAN2-R	5'- GCTTGTTCCCGAGATGCTTG -3'
KAN3-F	5'- TGAGAATGAATGGAAGAATCGAAG -3'
KAN3-R	5'- GGTCTTGTCCCTGTAAGGCTTG -3'
KAN4-F	5'- AGGATACCAACATTCATCTCAATTTG -3'
KAN4-R	5'- CACTGGAGGGTTCCGCATATT -3'
FIL-F	5'- AAACCAACATGCCCCAACAG -3'
FIL-R	5'- TCACACCAACGTTAGCAGCTG -3'
YAB2-F	5'- TTGGGCACATTTTCCTCACA -3'
YAB2-R	5'- GCAACTGACTGGTCTAATTGCTTG -3'
YAB3-F	5'- CCATTCCTTTTTACCTCCCCC -3'
YAB3-R	5'- TTTATATTCTGCCCTCCGCTTC -3'
YAB5-F	5'- ACGCCCTAATTTCCAGGCAAC -3'
YAB5-R	5'- GTTGCTCAGTTATGGTACGAG -3'
ETT-F	5'- CGCCTACTCAATAACCGATCATC -3'
ETT-R	5'- ACGGCCCACACCAAATGTT -3'
ARF4-F	5'- CGCTTAAATCATTCCCGCAAT -3'
ARF4-R	5'- ACTTGTTGGCTTGGTAAGCAAAG -3'
PHB-F	5'- GCTGTTGACTGGGTTCAGATGA -3'
PHB-R	5'- GCGAAATAGCGACTATGCCAAT -3'
PHV-F	5'- GGCGGAGTTCCTTTGCAA -3'
PHV-R	5'- CCAGGCTTCATCCCAATCAT -3'
REV-F	5'- ATCCAAAGTCGTTGCACAAAAA -3'
REV-R	5'- GACTCTTGGGCTAATTGCCTGAT -3'
TUA3-F	5'- GTGCTGAAGGTGGAGACGAT -3'
TUA3-R	5'- AACACGAAGACCGAACGAAT -3'

Supplementary Table S2. Changes in transcription levels of class 1 *KNOX* genes and genes related to determination of leaf adaxial-abaxial polarity.

Each value was normalized by reference to the level of *TUA3* transcripts. Values and standard deviations (SD: n = 3) are shown relative to the value for wild-type plants treated with 0 μM berberine. * and ** indicate significant difference from the value for each plant not treated with berberine (P < 0.05 and P < 0.01 by Student's T-test, respectively).

gene	berberine (μM)	wild type			as1-1			as2-1		
		mean	±	SD	mean	±	SD	mean	±	SD
Class 1 *KNOX* genes										
BREVIPEDICELLUS	0	1.0	±	0.1	2.6	±	0.2	2.4	±	0.2
	5	2.1**	±	0.1	6.8**	±	0.4	2.9*	±	0.2
	12.5	2.6**	±	0.3	7.3**	±	0.5	3.7*	±	0.7
KNAT2	0	1.0	±	0.1	1.3	±	0.1	1.3	±	0.5
	5	1.3	±	0.2	2.5*	±	0.4	1.7	±	0.5
	12.5	1.4*	±	0.2	2.7*	±	0.4	2.6	±	1.2
KNAT6	0	1.0	±	0.1	1.2	±	0.2	1.2	±	0.1
	5	1.7*	±	0.2	2.3**	±	0.2	1.4	±	0.2
	12.5	1.5	±	0.4	2.7**	±	0.1	2.4**	±	0.2
SHOOT MERISTEMLESS	0	1.0	±	0.2	0.7	±	0.0	1.0	±	0.2
	5	1.2	±	0.3	1.0	±	0.8	1.2	±	0.4
	12.5	0.9	±	0.2	1.6*	±	0.2	1.3	±	0.1
KANADI genes										
KANADI1	0	1.0	±	0.1	0.8	±	0.0	1.1	±	0.1
	5	0.9	±	0.0	1.1**	±	0.1	1.0	±	0.2
	12.5	1.0	±	0.1	1.2**	±	0.1	1.5*	±	0.1
KANADI2	0	1.0	±	0.2	1.1	±	0.1	1.4	±	0.1
	5	1.3	±	0.2	1.4	±	0.1	1.3	±	0.2
	12.5	1.0	±	0.1	1.9*	±	0.3	2.2**	±	0.2
KANADI3	0	1.0	±	0.2	0.5	±	0.3	1.0	±	0.1
	5	1.4	±	0.2	1.1	±	0.2	1.5	±	0.3
	12.5	1.8**	±	0.2	1.5*	±	0.1	2.1	±	0.8
KANADI4	0	1.0	±	0.2	0.6	±	0.1	1.1	±	0.3
	5	1.4	±	0.1	1.7**	±	0.1	1.5	±	0.2
	12.5	2.0**	±	0.2	2.0**	±	0.3	2.7*	±	0.7

Supplementary Table S2. Continued.

gene	berberine (μM)	wild type			*as1-1*			*as2-1*		
		mean	±	SD	mean	±	SD	mean	±	SD
YABBY genes										
FILAMENTOUS FLOWER	0	1.0	±	0.1	0.9	±	0.1	1.2	±	0.1
	5	1.2	±	0.2	1.2	±	0.0	1.5	±	0.1
	12.5	0.9	±	0.0	1.5*	±	0.2	2.0**	±	0.2
YABBY2	0	1.0	±	0.1	0.7	±	0.1	1.1	±	0.0
	5	0.8	±	0.1	0.9	±	0.0	0.9	±	0.1
	12.5	0.5*	±	0.0	1.0	±	0.2	1.3	±	0.1
YABBY3	0	1.0	±	0.3	0.7	±	0.1	1.2	±	0.1
	5	1.2	±	0.1	1.0	±	0.1	1.3	±	0.3
	12.5	1.0	±	0.1	1.2**	±	0.1	1.8*	±	0.2
YABBY5	0	1.0	±	0.2	1.2	±	0.1	2.2	±	0.2
	5	0.8	±	0.1	1.8**	±	0.0	2.2	±	0.2
	12.5	0.8	±	0.1	2.1**	±	0.1	3.3*	±	0.4
AUXIN RESPONSE FACTOR genes										
ETTIN/AUXIN RESPONSE FACTOR3	0	1.0	±	0.2	1.0	±	0.1	1.3	±	0.0
	5	1.4*	±	0.2	1.5**	±	0.1	1.5	±	0.2
	12.5	1.2	±	0.0	2.1**	±	0.2	2.5*	±	0.4
AUXIN RESPONSE FACTOR4	0	1.0	±	0.1	0.6	±	0.0	1.1	±	0.1
	5	1.4*	±	0.1	1.6**	±	0.1	1.5**	±	0.1
	12.5	1.0	±	0.2	1.6**	±	0.1	1.9**	±	0.1
HD-ZIP III genes										
PHABULOSA	0	1.0	±	0.2	0.6	±	0.1	0.9	±	0.1
	5	1.2	±	0.1	1.1*	±	0.0	0.9	±	0.1
	12.5	1.3	±	0.0	1.3**	±	0.2	1.4*	±	0.1
PHAVOLUTA	0	1.0	±	0.1	0.5	±	0.1	1.0	±	0.0
	5	1.0	±	0.1	1.0**	±	0.0	0.9	±	0.1
	12.5	1.0	±	0.1	1.1**	±	0.1	1.1	±	0.2
REVOLUTA	0	1.0	±	0.2	0.7	±	0.1	0.8	±	0.1
	5	1.2	±	0.1	1.1*	±	0.0	0.9	±	0.2
	12.5	1.1	±	0.1	1.1*	±	0.1	0.9	±	0.0

Supplementary Table S3.
AGI codes of genes upregulated or downregulated by treatment with berberine. AGI codes of genes and *n*-fold change (log2) are listed.

AGI code	*n*-fold change (log2)	AGI code	*n*-fold change (log2)	AGI code	*n*-fold change (log2)	AGI code	*n*-fold change (log2)
At5g04150	7.4	At5g62480	3.9	At3g44690	3.3	At5g61160	2.9
At1g47400	6.8	At2g30750	3.8	At5g58310	3.3	At3g18650	2.9
At1g12030	6.4	At2g32740	3.8	At5g61120	3.3	At2g42480	2.8
At3g60140	5.7	At1g52000	3.8	At2g38250	3.2	At1g61440	2.8
At2g23500	5.6	At1g52040	3.8	At1g32940	3.2	At1g50340	2.8
At2g36750	5.4	At4g37370	3.8	At1g02530	3.2	At1g48095	2.8
At2g16010	5.3	At3g46660	3.8	At1g71530	3.2	At3g28270	2.8
At5g59310	5.3	At5g17220	3.8	At3g23770	3.2	At3g61120	2.8
At1g05680	5.1	At2g27690	3.7	At3g61760	3.2	At5g25530	2.8
At2g36790	5.0	At2g21640	3.7	At5g07540	3.2	At2g23040	2.7
At2g04050	5.0	At1g36430	3.7	At5g43450	3.2	At2g27240	2.7
At2g41730	5.0	At1g63280	3.7	At5g53450	3.2	At2g17740	2.7
At3g27510	5.0	At3g28210	3.7	At2g35970	3.1	At2g37030	2.7
At3g25200	4.9	At1g22275	3.7	At1g14950	3.1	At1g64500	2.7
At3g55680	4.6	At5g05250	3.7	At4g07380	3.1	At1g32350	2.7
At5g44850	4.6	At5g58630	3.7	At4g21680	3.1	At3g16360	2.7
At1g46336	4.6	At2g18720	3.6	At3g28610	3.1	At3g24250	2.7
At2g04070	4.5	At2g15610	3.6	At1g10585	3.0	At3g50930	2.7
At3g56980	4.5	At1g26420	3.6	At1g17180	3.0	At3g51560	2.7
At2g46830	4.4	At5g09370	3.6	At1g50530	3.0	At3g53040	2.7
At2g04730	4.4	At2g30770	3.5	At3g22250	3.0	At3g61630	2.7
At3g32130	4.4	At2g42540	3.5	At4g22030	3.0	At3g09330	2.7
At2g32020	4.3	At3g02310	3.5	At3g51590	3.0	At2g33010	2.6
At3g28220	4.3	At4g11730	3.5	At5g67370	3.0	At2g15660	2.6
At1g28170	4.3	At3g56360	3.5	At5g24780	3.0	At1g30700	2.6
At2g44460	4.2	At5g40990	3.5	At2g38240	2.9	At3g19270	2.6
At1g11610	4.2	At2g20720	3.4	At2g38520	2.9	At4g01640	2.6
At3g46750	4.2	At1g34290	3.4	At2g36770	2.9	At4g04490	2.6
At5g49690	4.2	At1g34545	3.4	At2g46360	2.9	At4g23680	2.6
At5g58400	4.2	At1g17545	3.4	At2g27160	2.9	At3g60110	2.6
At1g51480	4.1	At3g05400	3.4	At4g03970	2.9	At5g06490	2.6
At1g68380	4.1	At3g01830	3.4	At1g77730	2.9	At5g09570	2.6
At3g54520	4.1	At3g03240	3.4	At3g22370	2.9	At5g42290	2.6
At2g03760	4.0	At4g26950	3.4	At3g15605	2.9	At5g55240	2.6
At3g06630	4.0	At3g49580	3.4	At3g10930	2.9	At5g58810	2.6
At3g21720	4.0	At5g15590	3.4	At4g08610	2.9	At4g16370	2.6
At4g09860	4.0	At2g31460	3.3	At3g42110	2.9	At2g30760	2.5
At4g18000	4.0	At1g18710	3.3	At3g43280	2.9	At2g47000	2.5
At4g26340	4.0	At1g72540	3.3	At3g45140	2.9	At2g29350	2.5
At5g58770	3.9	At3g11080	3.3	At5g51440	2.9	At2g02270	2.5

Supplementary Table S3. Continued.

AGI code	*n*-fold change (log2)	AGI code	*n*-fold change (log2)	AGI code	*n*-fold change (log2)	AGI code	*n*-fold change (log2)
At2g05980	2.5	At5g42830	2.2	At3g01970	1.9	At2g15480	1.7
At2g13870	2.5	At5g20230	2.2	At3g12660	1.9	At1g60730	1.7
At2g21420	2.5	At2g17000	2.2	At3g18290	1.9	At1g09380	1.7
At2g22160	2.5	At2g28820	2.1	At1g75290	1.9	At1g14250	1.7
At1g76470	2.5	At2g42410	2.1	At1g51670	1.9	At1g72900	1.7
At1g56430	2.5	At2g35950	2.1	At4g10370	1.9	At1g73040	1.7
At1g43910	2.5	At1g62763	2.1	At4g27030	1.9	At1g02850	1.7
At4g02740	2.5	At1g69150	2.1	At3g44040	1.9	At1g32870	1.7
At5g48130	2.5	At3g15500	2.1	At5g37560	1.9	At1g63350	1.7
At5g48850	2.5	At1g30135	2.1	At5g26340	1.9	At1g77570	1.7
At5g57690	2.5	At4g07600	2.1	At5g04180	1.9	At1g12550	1.7
At5g17130	2.5	At4g23600	2.1	At4g15530	1.9	At3g08860	1.7
At1g58390	2.5	At5g02900	2.1	At1g59265	1.9	At3g14620	1.7
At2g25565	2.4	At5g45120	2.1	At2g33710	1.8	At1g66550	1.7
At1g17170	2.4	At5g60070	2.1	At2g29470	1.8	At3g31023	1.7
At1g68620	2.4	At5g16860	2.1	At2g29460	1.8	At4g22470	1.7
At1g78410	2.4	At2g26560	2.1	At1g23020	1.8	At3g56080	1.7
At4g11230	2.4	At2g29490	2.0	At1g10170	1.8	At3g59610	1.7
At4g25810	2.4	At1g02200	2.0	At2g21820	1.8	At3g60650	1.7
At3g45730	2.4	At2g03580	2.0	At2g21320	1.8	At5g05600	1.7
At5g63740	2.4	At2g10070	2.0	At2g13630	1.8	At5g36910	1.7
At5g04190	2.4	At2g04040	2.0	At1g48300	1.8	At5g48270	1.7
At2g15490	2.3	At1g75750	2.0	At2g43820	1.8	At5g53510	1.7
At1g23730	2.3	At1g70910	2.0	At1g69930	1.8	At5g54060	1.7
At2g15410	2.3	At1g63200	2.0	At1g29430	1.8	At5g55800	1.7
At1g08270	2.3	At1g35310	2.0	At1g52400	1.8	At5g57220	1.7
At1g69880	2.3	At3g09520	2.0	At1g15890	1.8	At5g59320	1.7
At3g20030	2.3	At3g21670	2.0	At1g02340	1.8	At5g62800	1.7
At4g11760	2.3	At3g23550	2.0	At3g18250	1.8	At5g16980	1.7
At4g21120	2.3	At4g26290	2.0	At3g25670	1.8	At1g35140	1.7
At3g45270	2.3	At5g48490	2.0	At5g54100	1.8	At1g23550	1.7
At5g14730	2.3	At5g67000	2.0	At4g31870	1.8	At2g18680	1.6
At5g15850	2.3	At5g29958	2.0	At5g24160	1.8	At1g05060	1.6
At5g21120	2.3	At5g33370	2.0	At5g36770	1.8	At1g24090	1.6
At2g32540	2.2	At2g38340	1.9	At5g39850	1.8	At1g43160	1.6
At1g43290	2.2	At2g39030	1.9	At5g50800	1.8	At2g22030	1.6
At1g80130	2.2	At2g18050	1.9	At5g61730	1.8	At2g01860	1.6
At3g21170	2.2	At2g20670	1.9	At5g26220	1.8	At1g59790	1.6
At1g66630	2.2	At1g52100	1.9	At1g31820	1.8	At1g72920	1.6
At4g28280	2.2	At2g18180	1.9	At1g57780	1.8	At1g59590	1.6
At5g39050	2.2	At1g53870	1.9	At1g56650	1.8	At1g07500	1.6
At5g42800	2.2	At1g27730	1.9	At2g37770	1.7	At1g26380	1.6

Supplementary Table S3. Continued.

AGI code	*n*-fold change (log2)	AGI code	*n*-fold change (log2)	AGI code	*n*-fold change (log2)	AGI code	*n*-fold change (log2)
At1g21390	1.6	At1g71140	1.5	At1g42990	1.4	At2g04039	1.3
At1g74670	1.6	At1g02390	1.5	At1g28030	1.4	At1g65190	1.3
At1g35970	1.6	At3g04200	1.5	At3g03840	1.4	At1g59700	1.3
At1g36370	1.6	At3g15780	1.5	At3g03670	1.4	At1g28610	1.3
At3g05690	1.6	At3g25250	1.5	At3g04000	1.4	At1g17020	1.3
At3g02380	1.6	At1g66760	1.5	At3g15650	1.4	At1g77990	1.3
At3g25760	1.6	At1g20350	1.5	At3g27690	1.4	At1g21130	1.3
At3g28740	1.6	At2g33380	1.5	At3g16800	1.4	At1g80440	1.3
At1g19670	1.6	At4g04480	1.5	At3g12320	1.4	At1g29500	1.3
At1g16770	1.6	At4g12490	1.5	At4g09350	1.4	At1g29460	1.3
At4g02160	1.6	At4g19170	1.5	At4g19420	1.4	At1g21220	1.3
At4g03700	1.6	At3g56200	1.5	At4g19470	1.4	At1g13810	1.3
At4g04830	1.6	At5g12020	1.5	At4g21940	1.4	At3g01430	1.3
At4g08870	1.6	At5g24660	1.5	At4g23960	1.4	At3g08590	1.3
At4g34440	1.6	At5g40690	1.5	At4g24260	1.4	At3g04150	1.3
At4g38990	1.6	At5g42650	1.5	At4g27300	1.4	At3g29250	1.3
At3g62890	1.6	At5g56160	1.5	At4g28680	1.4	At3g19620	1.3
At5g11120	1.6	At5g59820	1.5	At4g31330	1.4	At1g75280	1.3
At5g52140	1.6	At5g65110	1.5	At4g34410	1.4	At3g12520	1.3
At5g59810	1.6	At5g25130	1.5	At3g44970	1.4	At1g07010	1.3
At5g25470	1.6	At5g25960	1.5	At3g47640	1.4	At1g07180	1.3
At5g28020	1.6	At5g16970	1.5	At3g48520	1.4	At1g17800	1.3
At2g26020	1.6	At5g19720	1.5	At3g48720	1.4	At4g03320	1.3
At2g30140	1.5	At4g14810	1.5	At5g13740	1.4	At4g21990	1.3
At2g23000	1.5	At4g17500	1.5	At5g48180	1.4	At4g22870	1.3
At2g37760	1.5	At1g44446	1.5	At5g64770	1.4	At4g28460	1.3
At2g25735	1.5	At2g29820	1.4	At5g65550	1.4	At4g39740	1.3
At2g29450	1.5	At2g38790	1.4	At5g66400	1.4	At3g48360	1.3
At2g29340	1.5	At2g18690	1.4	At5g27350	1.4	At3g50480	1.3
At1g03660	1.5	At2g32030	1.4	At1g73480	1.4	At3g55620	1.3
At1g54720	1.5	At1g63340	1.4	At4g14730	1.4	At3g63380	1.3
At2g42750	1.5	At1g27240	1.4	At1g29440	1.4	At5g13200	1.3
At2g31380	1.5	At2g07684	1.4	At2g39640	1.3	At5g18060	1.3
At1g64980	1.5	At1g64710	1.4	At2g26130	1.3	At5g18560	1.3
At1g75960	1.5	At1g19610	1.4	At2g35480	1.3	At5g24280	1.3
At1g02930	1.5	At1g17420	1.4	At2g15080	1.3	At5g42450	1.3
At1g80160	1.5	At1g21910	1.4	At2g35733	1.3	At5g43980	1.3
At1g05880	1.5	At1g06160	1.4	At2g42930	1.3	At5g46680	1.3
At1g32430	1.5	At2g43580	1.4	At2g40350	1.3	At5g52390	1.3
At1g69790	1.5	At2g43510	1.4	At2g01850	1.3	At5g53870	1.3
At1g68765	1.5	At1g68320	1.4	At2g42530	1.3	At5g55200	1.3
At1g76600	1.5	At1g69610	1.4	At2g04032	1.3	At5g55570	1.3

Supplementary Table S3. Continued.

AGI code	*n*-fold change (log2)	AGI code	*n*-fold change (log2)	AGI code	*n*-fold change (log2)	AGI code	*n*-fold change (log2)
At5g57560	1.3	At3g02150	1.2	At2g30250	1.1	At4g28160	1.1
At5g62280	1.3	At3g10830	1.2	At2g23900	1.1	At4g29030	1.1
At5g65730	1.3	At3g08810	1.2	At2g37970	1.1	At4g32950	1.1
At5g25190	1.3	At3g17010	1.2	At2g03370	1.1	At4g33663	1.1
At5g25610	1.3	At4g10240	1.2	At2g20870	1.1	At3g44300	1.1
At4g15440	1.3	At4g03500	1.2	At1g23740	1.1	At3g46670	1.1
At1g44414	1.3	At4g07880	1.2	At1g05290	1.1	At3g49320	1.1
At1g44350	1.3	At4g10910	1.2	At1g77210	1.1	At5g03860	1.1
At1g63580	1.3	At4g21080	1.2	At1g70270	1.1	At5g06920	1.1
At2g30790	1.2	At4g25780	1.2	At1g79370	1.1	At5g07100	1.1
At2g30520	1.2	At4g26200	1.2	At2g36080	1.1	At5g10140	1.1
At2g30510	1.2	At4g33666	1.2	At2g04080	1.1	At5g13360	1.1
At2g34810	1.2	At4g34250	1.2	At1g64700	1.1	At5g14105	1.1
At2g03310	1.2	At4g39510	1.2	At1g75910	1.1	At5g23060	1.1
At2g46680	1.2	At3g44480	1.2	At1g75900	1.1	At5g23940	1.1
At2g24100	1.2	At3g47340	1.2	At1g52700	1.1	At5g37150	1.1
At2g37240	1.2	At3g49620	1.2	At1g33110	1.1	At5g43580	1.1
At2g35740	1.2	At3g54150	1.2	At1g33130	1.1	At5g46050	1.1
At2g05540	1.2	At5g02940	1.2	At1g07430	1.1	At5g49520	1.1
At1g22630	1.2	At5g03090	1.2	At1g17380	1.1	At5g55620	1.1
At1g31100	1.2	At5g03490	1.2	At1g70700	1.1	At5g56200	1.1
At2g18190	1.2	At5g03930	1.2	At1g76590	1.1	At5g59540	1.1
At1g23390	1.2	At5g06980	1.2	At1g75100	1.1	At5g60090	1.1
At1g75720	1.2	At5g24420	1.2	At1g71330	1.1	At5g62140	1.1
At1g50290	1.2	At5g39080	1.2	At1g29510	1.1	At5g67330	1.1
At1g02740	1.2	At5g40670	1.2	At1g19020	1.1	At5g16990	1.1
At1g22380	1.2	At5g46180	1.2	At3g01060	1.1	At3g44860	1.1
At1g22400	1.2	At5g47220	1.2	At3g26830	1.1	At5g13080	1.1
At1g80840	1.2	At5g50100	1.2	At3g21690	1.1	At1g73360	1.1
At1g76070	1.2	At5g56100	1.2	At3g23560	1.1	At1g28230	1.1
At1g18810	1.2	At5g57345	1.2	At3g16220	1.1	At4g17030	1.1
At1g33030	1.2	At5g57550	1.2	At3g17770	1.1	At4g15630	1.1
At1g32920	1.2	At5g64870	1.2	At3g14450	1.1	At2g22990	1.0
At1g26390	1.2	At5g64850	1.2	At3g28290	1.1	At2g32510	1.0
At1g78160	1.2	At5g25240	1.2	At1g72060	1.1	At2g23945	1.0
At1g67980	1.2	At5g26270	1.2	At3g12460	1.1	At2g41250	1.0
At1g76690	1.2	At5g27420	1.2	At4g11930	1.1	At2g29320	1.0
At1g29450	1.2	At5g28090	1.2	At4g18210	1.1	At2g29420	1.0
At1g52410	1.2	At1g48910	1.2	At4g20860	1.1	At2g27550	1.0
At1g48000	1.2	At2g38150	1.2	At4g21620	1.1	At2g39100	1.0
At1g35350	1.2	At1g33840	1.2	At4g24660	1.1	At2g38870	1.0
At3g03830	1.2	At1g55050	1.2	At4g28120	1.1	At2g15050	1.0

Supplementary Table S3. Continued.

AGI code	*n*-fold change (log2)
At2g48020	1.0
At2g32100	1.0
At2g20800	1.0
At2g18300	1.0
At2g14080	1.0
At1g24650	1.0
At1g23130	1.0
At1g08920	1.0
At1g10370	1.0
At1g43230	1.0
At2g25200	1.0
At1g67520	1.0
At1g02205	1.0
At2g40200	1.0
At1g54120	1.0
At1g23240	1.0
At1g28600	1.0
At1g16290	1.0
At1g50400	1.0
At1g49500	1.0
At1g72940	1.0
At1g64170	1.0
At1g77960	1.0
At1g52590	1.0
At1g18870	1.0
At1g79680	1.0
At1g02660	1.0
At1g21410	1.0
At1g15010	1.0
At1g72460	1.0
At1g72450	1.0
At1g73870	1.0
At1g68780	1.0
At1g29490	1.0
At3g05110	1.0
At3g03580	1.0
At3g06070	1.0
At3g26940	1.0
At3g26220	1.0
At3g26280	1.0
At3g30720	1.0
At3g19000	1.0

AGI code	*n*-fold change (log2)
At1g66130	1.0
At1g75270	1.0
At1g33430	1.0
At1g19150	1.0
At4g02380	1.0
At4g08290	1.0
At4g13800	1.0
At4g18360	1.0
At4g21510	1.0
At4g21910	1.0
At4g28270	1.0
At4g28290	1.0
At4g34940	1.0
At4g35090	1.0
At4g38690	1.0
At3g43270	1.0
At3g45300	1.0
At3g48020	1.0
At3g49300	1.0
At3g55130	1.0
At3g56290	1.0
At3g57700	1.0
At3g59940	1.0
At5g03030	1.0
At5g05190	1.0
At5g10420	1.0
At5g12050	1.0
At5g13330	1.0
At5g18290	1.0
At5g23210	1.0
At5g37230	1.0
At5g38900	1.0
At5g39620	1.0
At5g43870	1.0
At5g44980	1.0
At5g45820	1.0
At5g47330	1.0
At5g53980	1.0
At5g56350	1.0
At5g57785	1.0
At5g58210	1.0
At5g61290	1.0

AGI code	*n*-fold change (log2)
At5g61820	1.0
At5g64840	1.0
At5g65380	1.0
At5g27360	1.0
At5g20250	1.0
At5g09290	1.0
At1g57990	1.0
At4g16563	1.0
At2g04020	1.0
At2g42280	0.9
At2g41870	0.9
At2g41120	0.9
At2g26870	0.9
At2g19800	0.9
At2g46100	0.9
At2g46220	0.9
At2g29300	0.9
At2g39120	0.9
At2g39050	0.9
At2g42430	0.9
At2g18010	0.9
At2g01380	0.9
At2g32090	0.9
At2g27390	0.9
At2g25510	0.9
At2g25450	0.9
At2g43000	0.9
At2g28400	0.9
At2g43010	0.9
At1g51210	0.9
At2g17910	0.9
At1g08900	0.9
At1g05340	0.9
At1g30795	0.9
At1g10150	0.9
At1g70290	0.9
At1g70420	0.9
At1g65450	0.9
At1g78930	0.9
At1g04350	0.9
At2g16990	0.9
At1g03070	0.9

AGI code	*n*-fold change (log2)
At1g03055	0.9
At2g05390	0.9
At1g06570	0.9
At1g64220	0.9
At1g70810	0.9
At1g65850	0.9
At1g15600	0.9
At1g06100	0.9
At1g19410	0.9
At1g36380	0.9
At1g72680	0.9
At1g69160	0.9
At3g06780	0.9
At3g15370	0.9
At3g14415	0.9
At3g14020	0.9
At3g14050	0.9
At3g13040	0.9
At3g26630	0.9
At3g27110	0.9
At3g30440	0.9
At3g21420	0.9
At3g13770	0.9
At3g28330	0.9
At1g13700	0.9
At1g19180	0.9
At1g22360	0.9
At2g33420	0.9
At3g29575	0.9
At4g03510	0.9
At4g04840	0.9
At4g09760	0.9
At4g11360	0.9
At4g17840	0.9
At4g18740	0.9
At4g18910	0.9
At4g21210	0.9
At4g21580	0.9
At4g23290	0.9
At4g27410	0.9
At4g33110	0.9
At4g35770	0.9

Supplementary Table S3. Continued.

AGI code	*n*-fold change (log2)	AGI code	*n*-fold change (log2)	AGI code	*n*-fold change (log2)	AGI code	*n*-fold change (log2)
At4g36030	0.9	At5g65850	0.9	At2g21210	0.8	At3g06500	0.8
At4g37295	0.9	At5g25930	0.9	At2g36460	0.8	At3g06760	0.8
At4g37910	0.9	At5g27800	0.9	At2g21330	0.8	At3g02610	0.8
At4g38540	0.9	At5g15000	0.9	At1g60030	0.8	At3g17930	0.8
At4g38840	0.9	At5g15950	0.9	At1g09310	0.8	At3g23580	0.8
At4g39235	0.9	At5g17000	0.9	At2g24860	0.8	At3g14700	0.8
At4g39800	0.9	At1g57770	0.9	At2g03980	0.8	At3g21270	0.8
At3g42820	0.9	At4g36500	0.9	At2g36145	0.8	At3g27050	0.8
At3g44320	0.9	At1g32220	0.9	At1g54100	0.8	At3g12800	0.8
At3g44500	0.9	At1g73500	0.9	At1g54160	0.8	At3g28080	0.8
At3g48990	0.9	At1g28190	0.9	At1g03020	0.8	At3g19580	0.8
At3g53690	0.9	At4g14680	0.9	At2g05380	0.8	At3g26450	0.8
At3g55110	0.9	At3g29810	0.9	At1g14050	0.8	At3g25690	0.8
At3g57680	0.9	At3g25950	0.9	At1g50250	0.8	At3g25610	0.8
At3g61210	0.9	At3g28090	0.9	At1g47610	0.8	At2g34070	0.8
At3g61220	0.9	At2g30600	0.8	At1g70830	0.8	At1g31580	0.8
At3g61890	0.9	At2g02710	0.8	At1g68660	0.8	At4g18440	0.8
At3g62550	0.9	At2g44130	0.8	At1g74930	0.8	At2g40610	0.8
At5g03230	0.9	At2g37540	0.8	At1g02940	0.8	At4g03400	0.8
At5g06190	0.9	At2g39230	0.8	At1g80180	0.8	At4g04010	0.8
At5g06510	0.9	At2g18820	0.8	At1g35550	0.8	At4g04620	0.8
At5g13770	0.9	At2g02930	0.8	At1g64680	0.8	At4g05020	0.8
At5g17200	0.9	At2g46790	0.8	At1g64660	0.8	At4g08250	0.8
At5g22270	0.9	At2g29670	0.8	At1g76100	0.8	At4g09650	0.8
At5g41050	0.9	At2g46250	0.8	At1g50020	0.8	At4g11330	0.8
At5g42750	0.9	At2g26980	0.8	At1g01060	0.8	At4g13010	0.8
At5g43170	0.9	At2g27830	0.8	At1g26800	0.8	At4g20200	0.8
At5g43330	0.9	At2g28120	0.8	At1g26700	0.8	At4g21870	0.8
At5g44680	0.9	At2g45170	0.8	At1g32840	0.8	At4g22890	0.8
At5g46310	0.9	At2g40720	0.8	At1g12880	0.8	At4g24350	0.8
At5g49450	0.9	At2g07724	0.8	At1g12250	0.8	At4g24380	0.8
At5g54890	0.9	At2g18490	0.8	At1g29195	0.8	At4g25940	0.8
At5g57420	0.9	At2g18590	0.8	At2g43530	0.8	At4g31010	0.8
At5g59130	0.9	At2g17972	0.8	At1g69310	0.8	At4g30993	0.8
At5g59750	0.9	At2g27500	0.8	At1g74550	0.8	At4g31710	0.8
At5g60730	0.9	At2g15960	0.8	At1g72140	0.8	At4g31800	0.8
At5g61370	0.9	At2g37150	0.8	At1g63090	0.8	At4g33660	0.8
At5g61640	0.9	At1g08610	0.8	At1g44000	0.8	At4g36040	0.8
At5g61890	0.9	At1g61430	0.8	At3g05165	0.8	At4g37770	0.8
At5g62430	0.9	At2g25080	0.8	At3g10520	0.8	At4g38330	0.8
At5g64490	0.9	At1g60260	0.8	At3g06483	0.8	At3g46600	0.8
At5g65010	0.9	At1g54740	0.8	At3g08690	0.8	At3g48420	0.8

Supplementary Table S3. Continued.

AGI code	*n*-fold change (log2)
At3g48690	0.8
At3g48740	0.8
At3g50750	0.8
At3g50820	0.8
At3g53250	0.8
At3g53210	0.8
At3g55740	0.8
At3g57380	0.8
At3g57260	0.8
At3g57440	0.8
At3g57520	0.8
At3g57770	0.8
At3g59700	0.8
At3g59820	0.8
At3g61340	0.8
At3g61840	0.8
At3g62150	0.8
At3g62930	0.8
At5g01520	0.8
At5g02630	0.8
At5g03050	0.8
At5g05110	0.8
At5g06430	0.8
At5g06870	0.8
At5g06910	0.8
At5g09660	0.8
At5g12000	0.8
At5g14090	0.8
At5g14100	0.8
At5g15290	0.8
At5g16340	0.8
At5g22250	0.8
At5g35970	0.8
At5g38530	0.8
At5g39400	0.8
At5g39610	0.8
At5g43260	0.8
At5g43750	0.8
At5g51830	0.8
At5g52900	0.8
At5g54510	0.8
At5g57060	0.8

AGI code	*n*-fold change (log2)
At5g57720	0.8
At5g57860	0.8
At5g59515	0.8
At5g61590	0.8
At5g62790	0.8
At5g63470	0.8
At5g64170	0.8
At5g64940	0.8
At5g66960	0.8
At5g67300	0.8
At5g67480	0.8
At5g25210	0.8
At5g33290	0.8
At1g58180	0.8
At1g40871	0.8
At3g56710	0.8
At5g21050	0.8
At5g20140	0.8
At5g09420	0.8
At1g57830	0.8
At4g14460	0.8
At4g16990	0.8
At4g16000	0.8
At2g47520	0.8
At3g20880	0.8
At2g42040	0.7
At2g30550	0.7
At2g34860	0.7
At2g40000	0.7
At2g23590	0.7
At2g41180	0.7
At2g34500	0.7
At2g30010	0.7
At2g46270	0.7
At2g35260	0.7
At2g31060	0.7
At2g38530	0.7
At2g29370	0.7
At2g02210	0.7
At2g18780	0.7
At2g18700	0.7
At2g07739	0.7

AGI code	*n*-fold change (log2)
At2g07734	0.7
At2g24240	0.7
At2g25720	0.7
At2g01730	0.7
At2g01740	0.7
At2g32150	0.7
At2g20730	0.7
At2g18290	0.7
At2g07695	0.7
At2g07701	0.7
At2g43060	0.7
At1g62510	0.7
At1g55480	0.7
At1g03630	0.7
At1g62290	0.7
At1g62180	0.7
At1g65520	0.7
At1g08940	0.7
At1g10000	0.7
At2g31200	0.7
At2g03750	0.7
At2g35900	0.7
At2g21970	0.7
At2g21960	0.7
At2g36950	0.7
At2g04490	0.7
At2g04480	0.7
At2g10140	0.7
At1g31170	0.7
At1g04500	0.7
At2g16280	0.7
At2g24820	0.7
At2g28630	0.7
At2g15150	0.7
At1g10500	0.7
At1g10550	0.7
At1g05560	0.7
At1g03030	0.7
At2g16070	0.7
At1g23300	0.7
At1g75770	0.7
At1g79510	0.7

AGI code	*n*-fold change (log2)
At1g11600	0.7
At1g28570	0.7
At1g14130	0.7
At1g06430	0.7
At1g15260	0.7
At1g17240	0.7
At1g74710	0.7
At1g02860	0.7
At1g56050	0.7
At1g08320	0.7
At1g76190	0.7
At1g04770	0.7
At1g75460	0.7
At1g07280	0.7
At1g02620	0.7
At1g02475	0.7
At1g21400	0.7
At1g32290	0.7
At1g69890	0.7
At1g68530	0.7
At1g78200	0.7
At1g73680	0.7
At1g68010	0.7
At1g71480	0.7
At1g29660	0.7
At1g77670	0.7
At1g56510	0.7
At1g01500	0.7
At3g10525	0.7
At3g10500	0.7
At3g08000	0.7
At3g02730	0.7
At3g04120	0.7
At3g16660	0.7
At3g16570	0.7
At3g17615	0.7
At3g26750	0.7
At3g29035	0.7
At3g21790	0.7
At3g18450	0.7
At3g27350	0.7
At3g30775	0.7

Supplementary Table S3. Continued.

AGI code	*n*-fold change (log2)	AGI code	*n*-fold change (log2)	AGI code	*n*-fold change (log2)	AGI code	*n*-fold change (log2)
At3g21890	0.7	At3g47480	0.7	At5g16140	0.7	At1g65295	0.6
At3g15060	0.7	At3g51660	0.7	At5g28370	0.7	At2g31240	0.6
At3g15352	0.7	At3g51670	0.7	At5g11050	0.7	At2g42790	0.6
At3g19010	0.7	At3g53960	0.7	At5g13550	0.7	At2g36940	0.6
At1g69540	0.7	At3g54900	0.7	At1g33600	0.7	At2g36835	0.6
At3g12170	0.7	At3g56070	0.7	At5g04340	0.7	At2g40435	0.6
At1g55080	0.7	At3g56060	0.7	At4g15620	0.7	At1g60000	0.6
At1g34000	0.7	At3g59060	0.7	At4g15770	0.7	At1g31190	0.6
At1g22140	0.7	At3g62410	0.7	At4g17580	0.7	At1g04290	0.6
At1g22370	0.7	At5g01550	0.7	At4g14716	0.7	At2g04795	0.6
At1g16720	0.7	At5g03070	0.7	At2g40880	0.7	At2g22190	0.6
At4g00190	0.7	At5g03880	0.7	At2g26600	0.7	At1g78460	0.6
At4g00750	0.7	At5g07000	0.7	At2g26530	0.7	At2g05310	0.6
At4g01080	0.7	At5g13650	0.7	At1g66480	0.7	At1g23430	0.6
At4g03410	0.7	At5g14660	0.7	At2g45600	0.6	At1g64900	0.6
At4g03900	0.7	At5g17380	0.7	At2g26250	0.6	At1g14700	0.6
At4g04610	0.7	At5g22920	0.7	At2g19310	0.6	At1g13110	0.6
At4g09050	0.7	At5g24210	0.7	At2g23810	0.6	At1g43560	0.6
At4g09910	0.7	At5g37490	0.7	At2g34460	0.6	At1g43330	0.6
At4g12690	0.7	At5g38850	0.7	At2g39400	0.6	At1g76020	0.6
At4g18880	0.7	At5g39360	0.7	At2g26910	0.6	At1g14150	0.6
At4g22380	0.7	At5g39860	0.7	At2g44920	0.6	At1g06690	0.6
At4g22710	0.7	At5g41960	0.7	At2g02880	0.6	At1g72830	0.6
At4g23300	0.7	At5g45090	0.7	At2g46910	0.6	At1g53885	0.6
At4g24000	0.7	At5g52070	0.7	At2g07140	0.6	At1g02750	0.6
At4g25050	0.7	At5g54170	0.7	At2g01590	0.6	At1g66000	0.6
At4g27700	0.7	At5g54390	0.7	At2g27520	0.6	At1g50600	0.6
At4g28150	0.7	At5g57340	0.7	At2g27840	0.6	At1g18400	0.6
At4g29750	0.7	At5g57800	0.7	At2g02390	0.6	At1g18320	0.6
At4g30470	0.7	At5g58070	0.7	At2g45180	0.6	At1g18480	0.6
At4g31030	0.7	At5g62290	0.7	At2g24150	0.6	At1g01140	0.6
At4g31150	0.7	At5g64680	0.7	At2g19650	0.6	At1g01740	0.6
At4g32280	0.7	At5g65870	0.7	At2g03550	0.6	At1g01840	0.6
At4g33540	0.7	At5g66590	0.7	At2g05620	0.6	At1g07700	0.6
At4g33960	0.7	At5g67290	0.7	At2g16700	0.6	At1g18890	0.6
At4g37310	0.7	At5g25120	0.7	At1g55450	0.6	At1g44930	0.6
At4g37610	0.7	At5g27110	0.7	At1g24440	0.6	At1g48460	0.6
At4g37800	0.7	At5g27760	0.7	At2g17270	0.6	At1g26770	0.6
At3g43800	0.7	At1g48870	0.7	At1g22890	0.6	At1g12900	0.6
At3g45990	0.7	At5g15090	0.7	At1g65540	0.6	At1g12780	0.6
At3g45970	0.7	At5g16080	0.7	At1g09480	0.6	At1g53460	0.6
At3g47295	0.7	At5g16010	0.7	At1g27385	0.6	At1g53560	0.6

Supplementary Table S3. Continued.

AGI code	*n*-fold change (log2)	AGI code	*n*-fold change (log2)	AGI code	*n*-fold change (log2)	AGI code	*n*-fold change (log2)
At1g17290	0.6	At3g26170	0.6	At4g38970	0.6	At5g42850	0.6
At1g32520	0.6	At3g16520	0.6	At4g39950	0.6	At5g43850	0.6
At1g32500	0.6	At3g21090	0.6	At3g46360	0.6	At5g44190	0.6
At1g10900	0.6	At3g52340	0.6	At3g48000	0.6	At5g45350	0.6
At1g69935	0.6	At1g32550	0.6	At3g48200	0.6	At5g47880	0.6
At1g69210	0.6	At1g33490	0.6	At3g50250	0.6	At5g48590	0.6
At1g63880	0.6	At1g76890	0.6	At3g50740	0.6	At5g50410	0.6
At1g71970	0.6	At1g51780	0.6	At3g51510	0.6	At5g50720	0.6
At1g68830	0.6	At1g18060	0.6	At3g51920	0.6	At5g50920	0.6
At1g68820	0.6	At1g17920	0.6	At3g53280	0.6	At5g50960	0.6
At1g68020	0.6	At2g30330	0.6	At3g54120	0.6	At5g51190	0.6
At1g53670	0.6	At2g30270	0.6	At3g54140	0.6	At5g51390	0.6
At1g52200	0.6	At4g00238	0.6	At3g55040	0.6	At5g51970	0.6
At1g49830	0.6	At4g00790	0.6	At3g55560	0.6	At5g52450	0.6
At1g29410	0.6	At4g00780	0.6	At3g55820	0.6	At5g53820	0.6
At1g29700	0.6	At4g02410	0.6	At3g56010	0.6	At5g56180	0.6
At1g60990	0.6	At4g02530	0.6	At3g56940	0.6	At5g57040	0.6
At1g55370	0.6	At4g04020	0.6	At3g57190	0.6	At5g57710	0.6
At1g42980	0.6	At4g05440	0.6	At3g57360	0.6	At5g57950	0.6
At1g28140	0.6	At4g07940	0.6	At3g57780	0.6	At5g57900	0.6
At1g20510	0.6	At4g08780	0.6	At3g58180	0.6	At5g58140	0.6
At1g02370	0.6	At4g12500	0.6	At3g59400	0.6	At5g58330	0.6
At3g03890	0.6	At4g13180	0.6	At3g60180	0.6	At5g58690	0.6
At3g05200	0.6	At4g13195	0.6	At5g02050	0.6	At5g58870	0.6
At3g09050	0.6	At4g19490	0.6	At5g02120	0.6	At5g60120	0.6
At3g09010	0.6	At4g22530	0.6	At5g02710	0.6	At5g61220	0.6
At3g02240	0.6	At4g23670	0.6	At5g05690	0.6	At5g61520	0.6
At3g11690	0.6	At4g23880	0.6	At5g10070	0.6	At5g62020	0.6
At3g01440	0.6	At4g24340	0.6	At5g11840	0.6	At5g63900	0.6
At3g04730	0.6	At4g24700	0.6	At5g13400	0.6	At5g64780	0.6
At3g11930	0.6	At4g27830	0.6	At5g13750	0.6	At5g65710	0.6
At3g08020	0.6	At4g27990	0.6	At5g14120	0.6	At5g65720	0.6
At3g04520	0.6	At4g29430	0.6	At5g14410	0.6	At5g65840	0.6
At3g06080	0.6	At4g30530	0.6	At5g17230	0.6	At5g67030	0.6
At3g22310	0.6	At4g31390	0.6	At5g22290	0.6	At5g04830	0.6
At3g15450	0.6	At4g31790	0.6	At5g23750	0.6	At5g29080	0.6
At3g17890	0.6	At4g33550	0.6	At5g24010	0.6	At5g14780	0.6
At3g14690	0.6	At4g34135	0.6	At5g24650	0.6	At5g15450	0.6
At3g28940	0.6	At4g34590	0.6	At5g36120	0.6	At5g17010	0.6
At3g19850	0.6	At4g38020	0.6	At5g36700	0.6	At3g44880	0.6
At3g13062	0.6	At4g37930	0.6	At5g40170	0.6	At1g31920	0.6
At3g27060	0.6	At4g38850	0.6	At5g42070	0.6	At4g36940	0.6

Supplementary Table S3. Continued.

AGI code	*n*-fold change (log2)
At4g37260	0.6
At5g20935	0.6
At5g19470	0.6
At5g19500	0.6
At5g28750	0.6
At5g09270	0.6
At1g32160	0.6
At1g51090	0.6
At5g04250	0.6
At1g25275	0.6
At4g16670	0.6
At4g15800	0.6
At1g58842	0.6
At2g26580	0.6
At1g24040	0.6
At3g18460	0.6
At2g42190	0.5
At2g26670	0.5
At2g30610	0.5
At2g33880	0.5
At2g33760	0.5
At2g34930	0.5
At2g44510	0.5
At2g30970	0.5
At2g38210	0.5
At2g39150	0.5
At2g39470	0.5
At2g34620	0.5
At2g45740	0.5
At2g22870	0.5
At2g46820	0.5
At2g19810	0.5
At2g29650	0.5
At2g35390	0.5
At2g14660	0.5
At2g01570	0.5
At2g06850	0.5
At2g37920	0.5
At2g24180	0.5
At2g01320	0.5
At2g27260	0.5
At2g06050	0.5

AGI code	*n*-fold change (log2)
At2g20940	0.5
At1g05010	0.5
At1g51400	0.5
At1g62630	0.5
At1g76940	0.5
At1g22985	0.5
At1g05140	0.5
At2g25140	0.5
At1g12020	0.5
At2g42710	0.5
At2g24540	0.5
At1g60010	0.5
At2g04700	0.5
At2g31750	0.5
At2g22260	0.5
At2g04980	0.5
At2g05070	0.5
At1g05575	0.5
At1g54110	0.5
At1g23360	0.5
At1g65810	0.5
At1g64970	0.5
At1g62730	0.5
At1g06650	0.5
At1g15290	0.5
At1g34180	0.5
At1g21830	0.5
At1g34760	0.5
At1g64105	0.5
At1g70820	0.5
At1g70760	0.5
At1g74880	0.5
At1g77930	0.5
At1g49930	0.5
At1g49975	0.5
At1g01630	0.5
At1g14280	0.5
At1g18730	0.5
At1g32830	0.5
At1g19660	0.5
At1g62960	0.5
At1g75410	0.5

AGI code	*n*-fold change (log2)
At1g01430	0.5
At1g12200	0.5
At1g12220	0.5
At1g53580	0.5
At1g06040	0.5
At1g21500	0.5
At1g48030	0.5
At1g62420	0.5
At1g55850	0.5
At1g55910	0.5
At2g43750	0.5
At2g43540	0.5
At2g41705	0.5
At1g51500	0.5
At1g72430	0.5
At1g69760	0.5
At1g70580	0.5
At1g63970	0.5
At1g74450	0.5
At1g52870	0.5
At1g78140	0.5
At1g68795	0.5
At1g76730	0.5
At1g52230	0.5
At1g72175	0.5
At1g71030	0.5
At1g42970	0.5
At1g12450	0.5
At1g19000	0.5
At1g01490	0.5
At3g03870	0.5
At3g01510	0.5
At3g10270	0.5
At3g10230	0.5
At3g07470	0.5
At3g08940	0.5
At3g10450	0.5
At3g10050	0.5
At3g11900	0.5
At3g09580	0.5
At3g07930	0.5
At3g06750	0.5

AGI code	*n*-fold change (log2)
At3g02470	0.5
At3g16610	0.5
At3g15770	0.5
At3g17800	0.5
At3g23605	0.5
At3g26070	0.5
At3g16250	0.5
At3g19480	0.5
At3g20820	0.5
At3g26710	0.5
At3g29240	0.5
At3g12685	0.5
At3g26140	0.5
At3g25740	0.5
At3g27870	0.5
At3g24430	0.5
At3g19170	0.5
At3g19030	0.5
At3g21360	0.5
At3g22130	0.5
At3g22160	0.5
At1g42550	0.5
At1g66160	0.5
At3g11170	0.5
At3g12370	0.5
At1g67050	0.5
At2g30290	0.5
At2g33390	0.5
At1g18620	0.5
At1g18570	0.5
At1g25520	0.5
At4g02075	0.5
At4g02920	0.5
At4g03070	0.5
At4g05070	0.5
At4g07450	0.5
At4g09900	0.5
At4g09890	0.5
At4g10060	0.5
At4g10960	0.5
At4g11570	0.5
At4g12310	0.5

Supplementary Table S3. Continued.

AGI code	*n*-fold change (log2)	AGI code	*n*-fold change (log2)	AGI code	*n*-fold change (log2)	AGI code	*n*-fold change (log2)
At4g12470	0.5	At3g51730	0.5	At5g52190	0.5	At2g02950	0.4
At4g12480	0.5	At3g52840	0.5	At5g58260	0.5	At2g47010	0.4
At4g13850	0.5	At3g53360	0.5	At5g60360	0.5	At2g23840	0.4
At4g17900	0.5	At3g53470	0.5	At5g61030	0.5	At2g47910	0.4
At4g19840	0.5	At3g53420	0.5	At5g62350	0.5	At2g31070	0.4
At4g21170	0.5	At3g54500	0.5	At5g62830	0.5	At2g41420	0.4
At4g22200	0.5	At3g55610	0.5	At5g63200	0.5	At2g01490	0.4
At4g24450	0.5	At3g55640	0.5	At5g64080	0.5	At2g29360	0.4
At4g24670	0.5	At3g56430	0.5	At5g04850	0.5	At2g29540	0.4
At4g25080	0.5	At3g57450	0.5	At5g27520	0.5	At2g07741	0.4
At4g25910	0.5	At3g57690	0.5	At5g15980	0.5	At3g32920	0.4
At4g25900	0.5	At3g60130	0.5	At5g16030	0.5	At2g19540	0.4
At4g26500	0.5	At3g61980	0.5	At1g57760	0.5	At2g05520	0.4
At4g27340	0.5	At3g62450	0.5	At4g36580	0.5	At2g15890	0.4
At4g27470	0.5	At3g62650	0.5	At4g37080	0.5	At2g15690	0.4
At4g28240	0.5	At3g63520	0.5	At5g19940	0.5	At2g20830	0.4
At4g28660	0.5	At5g02160	0.5	At5g19855	0.5	At2g20270	0.4
At4g29820	0.5	At5g02280	0.5	At5g19440	0.5	At1g67300	0.4
At4g30390	0.5	At5g02600	0.5	At5g08320	0.5	At1g03675	0.4
At4g31820	0.5	At5g03120	0.5	At5g11070	0.5	At1g23030	0.4
At4g31850	0.5	At5g03470	0.5	At1g35190	0.5	At1g22850	0.4
At4g32340	0.5	At5g03660	0.5	At1g66900	0.5	At2g17670	0.4
At4g34730	0.5	At5g04040	0.5	At1g73540	0.5	At1g61870	0.4
At4g34920	0.5	At5g04490	0.5	At1g51110	0.5	At1g78820	0.4
At4g35250	0.5	At5g05440	0.5	At1g51100	0.5	At1g60190	0.4
At4g35760	0.5	At5g06410	0.5	At4g14550	0.5	At1g79040	0.4
At4g38060	0.5	At5g06280	0.5	At4g14605	0.5	At2g36220	0.4
At4g38250	0.5	At5g07220	0.5	At4g16190	0.5	At2g21860	0.4
At4g39210	0.5	At5g08180	0.5	At4g16410	0.5	At2g21260	0.4
At3g45770	0.5	At5g08460	0.5	At4g15510	0.5	At1g26920	0.4
At3g46610	0.5	At5g10810	0.5	At4g16840	0.5	At2g31570	0.4
At3g46540	0.5	At5g11520	0.5	At4g15610	0.5	At1g21000	0.4
At3g47250	0.5	At5g13410	0.5	At4g16566	0.5	At1g16430	0.4
At3g47080	0.5	At5g16650	0.5	At4g14040	0.5	At1g13990	0.4
At3g47470	0.5	At5g18490	0.5	At2g33150	0.5	At1g23440	0.4
At3g47560	0.5	At5g24270	0.5	At2g47450	0.5	At1g47530	0.4
At3g47800	0.5	At5g38660	0.5	At2g30570	0.4	At1g70890	0.4
At3g50440	0.5	At5g40370	0.5	At2g23670	0.4	At1g42440	0.4
At3g50830	0.5	At5g40890	0.5	At2g32480	0.4	At1g37130	0.4
At3g51000	0.5	At5g44870	0.5	At2g45980	0.4	At1g15980	0.4
At3g51600	0.5	At5g44930	0.5	At2g45960	0.4	At1g08230	0.4
At3g51420	0.5	At5g49360	0.5	At2g26930	0.4	At1g76150	0.4

Supplementary Table S3. Continued.

AGI code	*n*-fold change (log2)	AGI code	*n*-fold change (log2)	AGI code	*n*-fold change (log2)	AGI code	*n*-fold change (log2)
At1g21065	0.4	At3g20700	0.4	At4g34180	0.4	At5g51020	0.4
At1g48450	0.4	At3g14770	0.4	At4g34350	0.4	At5g52100	0.4
At1g34440	0.4	At1g66100	0.4	At4g34540	0.4	At5g52970	0.4
At1g19740	0.4	At1g33270	0.4	At4g37530	0.4	At5g53020	0.4
At1g43670	0.4	At1g75220	0.4	At4g37980	0.4	At5g53330	0.4
At1g78340	0.4	At3g10970	0.4	At4g38700	0.4	At5g54080	0.4
At1g17510	0.4	At1g72050	0.4	At4g40045	0.4	At5g54500	0.4
At2g43560	0.4	At1g51805	0.4	At3g44620	0.4	At5g54580	0.4
At1g69870	0.4	At1g18170	0.4	At3g46530	0.4	At5g54540	0.4
At1g73880	0.4	At1g58360	0.4	At3g51490	0.4	At5g54940	0.4
At1g80510	0.4	At1g29910	0.4	At3g52155	0.4	At5g57330	0.4
At1g36390	0.4	At1g29850	0.4	At3g53630	0.4	At5g57815	0.4
At1g76520	0.4	At1g17990	0.4	At3g54360	0.4	At5g58920	0.4
At1g72180	0.4	At1g67070	0.4	At3g56630	0.4	At5g61360	0.4
At1g77450	0.4	At1g32060	0.4	At3g57090	0.4	At5g63800	0.4
At1g77440	0.4	At4g00895	0.4	At3g58170	0.4	At5g26770	0.4
At1g77710	0.4	At4g01280	0.4	At3g60370	0.4	At5g14740	0.4
At1g28100	0.4	At4g02770	0.4	At3g60690	0.4	At5g15640	0.4
At3g09085	0.4	At4g04950	0.4	At5g01650	0.4	At5g16930	0.4
At3g01550	0.4	At4g08920	0.4	At5g01880	0.4	At5g17560	0.4
At3g10210	0.4	At4g10330	0.4	At5g01750	0.4	At4g36530	0.4
At3g05410	0.4	At4g10920	0.4	At5g02760	0.4	At4g36400	0.4
At3g07310	0.4	At4g11960	0.4	At5g03900	0.4	At5g20900	0.4
At3g09390	0.4	At4g13220	0.4	At5g03970	0.4	At5g20180	0.4
At3g04760	0.4	At4g13530	0.4	At5g04790	0.4	At5g20130	0.4
At3g03990	0.4	At4g18010	0.4	At5g05750	0.4	At5g28840	0.4
At3g07910	0.4	At4g18040	0.4	At5g08050	0.4	At1g32170	0.4
At3g15460	0.4	At4g18975	0.4	At5g09590	0.4	At1g66890	0.4
At3g15850	0.4	At4g22000	0.4	At5g12170	0.4	At5g04230	0.4
At3g15840	0.4	At4g22220	0.4	At5g12890	0.4	At4g16210	0.4
At3g26085	0.4	At4g22920	0.4	At5g12950	0.4	At4g17700	0.4
At3g21250	0.4	At4g23410	0.4	At5g16400	0.4	At4g17810	0.4
At3g19290	0.4	At4g23400	0.4	At5g18850	0.4	At4g16520	0.4
At3g18820	0.4	At4g25890	0.4	At5g38510	0.4	At4g15420	0.4
At3g12780	0.4	At4g25960	0.4	At5g38980	0.4	At4g15545	0.4
At3g24190	0.4	At4g27390	0.4	At5g41210	0.4	At4g16770	0.4
At3g23710	0.4	At4g27710	0.4	At5g44020	0.4	At4g16760	0.4
At3g15260	0.4	At4g29190	0.4	At5g47370	0.4	At1g44575	0.4
At3g21020	0.4	At4g30270	0.4	At5g48030	0.4	At2g33210	0.4
At3g18980	0.4	At4g33640	0.4	At5g49230	0.4	At2g41650	0.4
At3g22850	0.4	At4g33650	0.4	At5g50250	0.4	At2g23170	0.4
At3g52220	0.4	At4g34020	0.4	At5g51070	0.4	At2g23290	0.4

Supplementary Table S3. Continued.

AGI code	*n*-fold change (log2)	AGI code	*n*-fold change (log2)	AGI code	*n*-fold change (log2)	AGI code	*n*-fold change (log2)
At2g26340	0.3	At1g15330	0.3	At4g04320	0.3	At5g45680	0.3
At2g23130	0.3	At1g17280	0.3	At4g04640	0.3	At5g46110	0.3
At2g30930	0.3	At1g33810	0.3	At4g05180	0.3	At5g47560	0.3
At2g38170	0.3	At1g08380	0.3	At4g05320	0.3	At5g48340	0.3
At2g14750	0.3	At1g79790	0.3	At4g19450	0.3	At5g51540	0.3
At2g34310	0.3	At1g19800	0.3	At4g21860	0.3	At5g53490	0.3
At2g34430	0.3	At1g26550	0.3	At4g22450	0.3	At5g54770	0.3
At2g35490	0.3	At1g43700	0.3	At4g23900	0.3	At5g56600	0.3
At2g47710	0.3	At2g47400	0.3	At4g24220	0.3	At5g57660	0.3
At2g27020	0.3	At1g73655	0.3	At4g24370	0.3	At5g58670	0.3
At2g27680	0.3	At1g72150	0.3	At4g27260	0.3	At5g60600	0.3
At2g02060	0.3	At1g19140	0.3	At4g27440	0.3	At5g61670	0.3
At2g02200	0.3	At1g13260	0.3	At4g28025	0.3	At5g61930	0.3
At2g07722	0.3	At3g07770	0.3	At4g29070	0.3	At5g62740	0.3
At2g24280	0.3	At3g01590	0.3	At4g29010	0.3	At5g63790	0.3
At2g42490	0.3	At3g04870	0.3	At4g30960	0.3	At5g63870	0.3
At2g03420	0.3	At3g07350	0.3	At4g32040	0.3	At5g64830	0.3
At2g03350	0.3	At3g06380	0.3	At4g34120	0.3	At5g65480	0.3
At2g27290	0.3	At3g08770	0.3	At4g37470	0.3	At5g15750	0.3
At2g20020	0.3	At3g06050	0.3	At4g38010	0.3	At5g16120	0.3
At2g16710	0.3	At3g23620	0.3	At3g45780	0.3	At5g16710	0.3
At2g36680	0.3	At3g27550	0.3	At3g46760	0.3	At5g17650	0.3
At1g24625	0.3	At3g42570	0.3	At3g47070	0.3	At3g51780	0.3
At1g27050	0.3	At3g12930	0.3	At3g47430	0.3	At3g51880	0.3
At1g67280	0.3	At3g26650	0.3	At3g50880	0.3	At4g36810	0.3
At1g61210	0.3	At3g18830	0.3	At3g51500	0.3	At5g32450	0.3
At1g23190	0.3	At3g29230	0.3	At3g52070	0.3	At5g20400	0.3
At1g22840	0.3	At3g26210	0.3	At3g52390	0.3	At5g21105	0.3
At1g27290	0.3	At3g26230	0.3	At3g52880	0.3	At1g58380	0.3
At1g78670	0.3	At3g23490	0.3	At3g54050	0.3	At1g47128	0.3
At1g54770	0.3	At3g26380	0.3	At3g57795	0.3	At1g25230	0.3
At2g36430	0.3	At3g13750	0.3	At3g62460	0.3	At4g14270	0.3
At2g21890	0.3	At3g32930	0.3	At3g63390	0.3	At4g14020	0.3
At2g13360	0.3	At1g66150	0.3	At5g02380	0.3	At4g16985	0.3
At1g54290	0.3	At1g75170	0.3	At5g02840	0.3	At4g14930	0.3
At1g64770	0.3	At1g07170	0.3	At5g05930	0.3	At1g67740	0.3
At1g64860	0.3	At1g18030	0.3	At5g13030	0.3	At1g67700	0.3
At1g11760	0.3	At4g10300	0.3	At5g19120	0.3	At1g71850	0.3
At1g16210	0.3	At1g16740	0.3	At5g22970	0.3	At2g38540	0.2
At1g14200	0.3	At4g01430	0.3	At5g23070	0.3	At2g20740	0.2
At1g13940	0.3	At4g01870	0.3	At5g23510	0.3	At1g55510	0.2
At1g06680	0.3	At4g03280	0.3	At5g37360	0.3	At1g61520	0.2

Supplementary Table S3. Continued.

AGI code	*n*-fold change (log2)	AGI code	*n*-fold change (log2)	AGI code	*n*-fold change (log2)	AGI code	*n*-fold change (log2)
At2g15380	0.2	At1g30220	0.2	At5g44260	-0.3	At1g54410	-0.4
At2g28605	0.2	At2g39730	0.2	At5g50000	-0.3	At1g75540	-0.4
At1g03090	0.2	At1g74470	0.1	At5g58040	-0.3	At1g79440	-0.4
At1g23980	0.2	At5g04590	0.1	At5g61970	-0.3	At1g74970	-0.4
At1g10740	0.2	At5g42860	0.1	At5g64060	-0.3	At1g22410	-0.4
At1g53850	0.2	At5g55210	0.1	At5g65670	-0.3	At1g10630	-0.4
At1g74730	0.2	At5g67220	0.1	At5g16130	-0.3	At1g15930	-0.4
At1g56170	0.2	At3g03060	0.0	At3g56880	-0.3	At1g08500	-0.4
At1g80190	0.2	At5g20370	0.0	At5g19430	-0.3	At1g08470	-0.4
At1g35513	0.2	At4g25380	-0.1	At2g47470	-0.3	At1g27760	-0.4
At1g11440	0.2	At2g42850	-0.2	At2g32810	-0.4	At1g49760	-0.4
At1g02560	0.2	At4g12330	-0.2	At2g02800	-0.4	At1g01090	-0.4
At1g74340	0.2	At5g50210	-0.2	At2g44180	-0.4	At1g71695	-0.4
At1g52930	0.2	At2g20760	-0.3	At2g19730	-0.4	At1g19960	-0.4
At1g71040	0.2	At1g09620	-0.3	At2g47930	-0.4	At1g06000	-0.4
At3g16370	0.2	At1g27400	-0.3	At2g27730	-0.4	At1g06890	-0.4
At3g09210	0.2	At1g22780	-0.3	At2g02450	-0.4	At1g19450	-0.4
At3g03980	0.2	At2g21580	-0.3	At2g37490	-0.4	At1g53240	-0.4
At3g26740	0.2	At1g10840	-0.3	At2g37270	-0.4	At1g78150	-0.4
At3g20330	0.2	At1g15120	-0.3	At2g01190	-0.4	At3g03780	-0.4
At3g15353	0.2	At1g80910	-0.3	At2g27510	-0.4	At3g09300	-0.4
At3g13520	0.2	At1g50560	-0.3	At2g21020	-0.4	At3g03250	-0.4
At3g12040	0.2	At1g73180	-0.3	At2g18230	-0.4	At3g07260	-0.4
At1g55000	0.2	At1g28130	-0.3	At2g22670	-0.4	At3g07100	-0.4
At1g20340	0.2	At3g01470	-0.3	At1g09970	-0.4	At3g07110	-0.4
At4g01890	0.2	At3g09840	-0.3	At1g09980	-0.4	At3g02570	-0.4
At4g12800	0.2	At3g09805	-0.3	At1g55760	-0.4	At3g15540	-0.4
At4g22260	0.2	At3g24550	-0.3	At1g77260	-0.4	At3g15430	-0.4
At4g28750	0.2	At3g29370	-0.3	At1g61740	-0.4	At3g23300	-0.4
At4g33010	0.2	At1g18210	-0.3	At1g11850	-0.4	At3g15610	-0.4
At4g34820	0.2	At4g11840	-0.3	At1g78920	-0.4	At3g17850	-0.4
At4g35000	0.2	At4g24920	-0.3	At1g67430	-0.4	At3g18035	-0.4
At3g54890	0.2	At4g29020	-0.3	At2g22510	-0.4	At3g18090	-0.4
At5g42980	0.2	At4g31920	-0.3	At2g35920	-0.4	At3g18080	-0.4
At5g43380	0.2	At3g46780	-0.3	At2g21390	-0.4	At3g28040	-0.4
At5g53880	0.2	At3g51720	-0.3	At1g26850	-0.4	At3g27190	-0.4
At5g54270	0.2	At3g55140	-0.3	At1g04310	-0.4	At3g28900	-0.4
At5g62050	0.2	At3g56240	-0.3	At1g04480	-0.4	At3g19120	-0.4
At5g35350	0.2	At5g02270	-0.3	At2g15240	-0.4	At3g13460	-0.4
At5g16060	0.2	At5g11970	-0.3	At2g04280	-0.4	At3g24040	-0.4
At5g20160	0.2	At5g22880	-0.3	At1g54080	-0.4	At3g23940	-0.4
At5g20620	0.2	At5g36880	-0.3	At2g27190	-0.4	At3g13772	-0.4

Supplementary Table S3. Continued.

AGI code	*n*-fold change (log2)
At3g29360	-0.4
At3g20000	-0.4
At3g11280	-0.4
At2g30260	-0.4
At1g25490	-0.4
At4g01310	-0.4
At4g01650	-0.4
At4g02680	-0.4
At4g13890	-0.4
At4g21650	-0.4
At4g22670	-0.4
At4g23850	-0.4
At4g24330	-0.4
At4g26530	-0.4
At4g27180	-0.4
At4g29680	-0.4
At4g29900	-0.4
At4g32720	-0.4
At4g34960	-0.4
At4g38660	-0.4
At4g39840	-0.4
At3g46640	-0.4
At3g50670	-0.4
At3g50950	-0.4
At3g52500	-0.4
At3g52750	-0.4
At3g54650	-0.4
At3g55460	-0.4
At3g57320	-0.4
At3g59660	-0.4
At3g60360	-0.4
At3g62820	-0.4
At5g03300	-0.4
At5g04750	-0.4
At5g06700	-0.4
At5g14430	-0.4
At5g14550	-0.4
At5g17910	-0.4
At5g22440	-0.4
At5g37290	-0.4
At5g37790	-0.4
At5g44920	-0.4

AGI code	*n*-fold change (log2)
At5g48450	-0.4
At5g48580	-0.4
At5g51560	-0.4
At5g52240	-0.4
At5g55100	-0.4
At5g55520	-0.4
At5g55550	-0.4
At5g56270	-0.4
At5g58950	-0.4
At5g59690	-0.4
At5g60920	-0.4
At5g61140	-0.4
At5g63400	-0.4
At5g63930	-0.4
At5g64090	-0.4
At5g64260	-0.4
At5g25460	-0.4
At5g27700	-0.4
At5g28060	-0.4
At5g35360	-0.4
At1g48860	-0.4
At5g14940	-0.4
At3g51770	-0.4
At4g36690	-0.4
At5g20885	-0.4
At1g28330	-0.4
At4g17560	-0.4
At4g14420	-0.4
At2g26660	-0.5
At2g45620	-0.5
At2g35020	-0.5
At2g44360	-0.5
At2g23120	-0.5
At2g38090	-0.5
At2g41110	-0.5
At2g34480	-0.5
At2g34585	-0.5
At2g46920	-0.5
At2g46225	-0.5
At2g47900	-0.5
At2g47730	-0.5
At2g47960	-0.5

AGI code	*n*-fold change (log2)
At2g46690	-0.5
At2g29550	-0.5
At2g40710	-0.5
At2g37340	-0.5
At2g15620	-0.5
At2g46530	-0.5
At1g03820	-0.5
At1g04040	-0.5
At1g24120	-0.5
At1g12000	-0.5
At1g03220	-0.5
At1g70370	-0.5
At1g02080	-0.5
At2g22450	-0.5
At2g36390	-0.5
At2g24570	-0.5
At2g01820	-0.5
At2g24940	-0.5
At1g78570	-0.5
At1g13170	-0.5
At1g47410	-0.5
At1g70770	-0.5
At1g48330	-0.5
At1g79920	-0.5
At1g64640	-0.5
At1g15910	-0.5
At1g18370	-0.5
At1g01960	-0.5
At1g49600	-0.5
At1g28440	-0.5
At1g07770	-0.5
At1g79700	-0.5
At1g48480	-0.5
At1g48600	-0.5
At1g12850	-0.5
At1g63000	-0.5
At1g29270	-0.5
At1g06760	-0.5
At2g47240	-0.5
At1g68490	-0.5
At1g79850	-0.5
At1g73230	-0.5

AGI code	*n*-fold change (log2)
At1g68810	-0.5
At1g67930	-0.5
At1g29350	-0.5
At3g11490	-0.5
At3g05530	-0.5
At3g02260	-0.5
At3g02250	-0.5
At3g03390	-0.5
At3g01810	-0.5
At3g06410	-0.5
At3g04570	-0.5
At3g09820	-0.5
At3g06590	-0.5
At3g02700	-0.5
At3g14720	-0.5
At3g21230	-0.5
At3g19820	-0.5
At3g18780	-0.5
At3g23510	-0.5
At3g13222	-0.5
At3g23690	-0.5
At3g26330	-0.5
At3g15190	-0.5
At3g22890	-0.5
At3g29180	-0.5
At3g52360	-0.5
At3g52200	-0.5
At3g11200	-0.5
At1g66470	-0.5
At3g12080	-0.5
At3g12120	-0.5
At1g58230	-0.5
At1g58370	-0.5
At1g66940	-0.5
At2g33310	-0.5
At4g00560	-0.5
At4g00720	-0.5
At4g00880	-0.5
At4g02470	-0.5
At4g03190	-0.5
At4g13250	-0.5
At4g19110	-0.5

Supplementary Table S3. Continued.

AGI code	*n*-fold change (log2)
At4g19160	-0.5
At4g22160	-0.5
At4g25150	-0.5
At4g25870	-0.5
At4g26130	-0.5
At4g27130	-0.5
At4g28190	-0.5
At4g29520	-0.5
At4g29700	-0.5
At4g30010	-0.5
At4g30200	-0.5
At4g32850	-0.5
At4g33580	-0.5
At4g34670	-0.5
At4g35750	-0.5
At4g35800	-0.5
At4g38320	-0.5
At4g38200	-0.5
At4g38520	-0.5
At4g38770	-0.5
At3g46970	-0.5
At3g48680	-0.5
At3g49120	-0.5
At3g49670	-0.5
At3g49530	-0.5
At3g53990	-0.5
At3g57330	-0.5
At3g60530	-0.5
At3g61490	-0.5
At2g47650	-0.5
At5g01020	-0.5
At5g05080	-0.5
At5g06610	-0.5
At5g10840	-0.5
At5g11340	-0.5
At5g11950	-0.5
At5g14040	-0.5
At5g17170	-0.5
At5g18410	-0.5
At5g24400	-0.5
At5g24490	-0.5
At5g35530	-0.5

AGI code	*n*-fold change (log2)
At5g41180	-0.5
At5g44240	-0.5
At5g44790	-0.5
At5g45500	-0.5
At5g47500	-0.5
At5g47910	-0.5
At5g51200	-0.5
At5g51820	-0.5
At5g52920	-0.5
At5g54800	-0.5
At5g56950	-0.5
At5g57870	-0.5
At5g60570	-0.5
At5g60640	-0.5
At5g61510	-0.5
At5g61900	-0.5
At5g62670	-0.5
At5g64130	-0.5
At5g65770	-0.5
At5g67360	-0.5
At5g67380	-0.5
At5g25560	-0.5
At5g26570	-0.5
At5g26707	-0.5
At5g26850	-0.5
At5g26830	-0.5
At5g16200	-0.5
At5g17160	-0.5
At4g36610	-0.5
At5g20950	-0.5
At5g19930	-0.5
At1g30270	-0.5
At4g14900	-0.5
At4g16500	-0.5
At4g16155	-0.5
At4g17190	-0.5
At1g55360	-0.5
At2g26640	-0.6
At2g26730	-0.6
At2g32730	-0.6
At2g45490	-0.6
At2g35000	-0.6

AGI code	*n*-fold change (log2)
At2g34300	-0.6
At2g34590	-0.6
At2g25930	-0.6
At2g29730	-0.6
At2g14900	-0.6
At2g14890	-0.6
At2g46240	-0.6
At2g06925	-0.6
At2g28790	-0.6
At2g45010	-0.6
At2g37850	-0.6
At2g38040	-0.6
At2g05920	-0.6
At2g05990	-0.6
At2g01630	-0.6
At2g17980	-0.6
At2g25490	-0.6
At2g37120	-0.6
At2g22660	-0.6
At2g07698	-0.6
At1g60810	-0.6
At2g17840	-0.6
At1g61450	-0.6
At1g55810	-0.6
At1g10060	-0.6
At1g11960	-0.6
At1g70300	-0.6
At1g60200	-0.6
At1g22770	-0.6
At1g78970	-0.6
At2g31320	-0.6
At2g35940	-0.6
At2g36350	-0.6
At2g46440	-0.6
At1g31180	-0.6
At2g25210	-0.6
At2g27150	-0.6
At2g20515	-0.6
At1g36060	-0.6
At1g65220	-0.6
At1g79530	-0.6
At1g59890	-0.6

AGI code	*n*-fold change (log2)
At1g20850	-0.6
At1g20950	-0.6
At1g11680	-0.6
At1g10760	-0.6
At1g76010	-0.6
At1g13950	-0.6
At1g15380	-0.6
At1g47630	-0.6
At1g64190	-0.6
At1g68560	-0.6
At1g78020	-0.6
At1g50490	-0.6
At1g76200	-0.6
At1g76090	-0.6
At1g47330	-0.6
At1g14290	-0.6
At1g79750	-0.6
At1g44800	-0.6
At1g35720	-0.6
At1g19600	-0.6
At1g01360	-0.6
At1g26440	-0.6
At1g53520	-0.6
At1g43710	-0.6
At1g19300	-0.6
At1g62330	-0.6
At1g53310	-0.6
At2g43800	-0.6
At1g10990	-0.6
At1g72400	-0.6
At1g74020	-0.6
At1g74030	-0.6
At1g76400	-0.6
At1g63050	-0.6
At1g20450	-0.6
At1g17720	-0.6
At3g16310	-0.6
At3g03640	-0.6
At3g04630	-0.6
At3g10810	-0.6
At3g08580	-0.6
At3g07990	-0.6

Supplementary Table S3. Continued.

AGI code	*n*-fold change (log2)	AGI code	*n*-fold change (log2)	AGI code	*n*-fold change (log2)	AGI code	*n*-fold change (log2)
At3g02750	-0.6	At4g13430	-0.6	At5g46240	-0.6	At2g35530	-0.7
At3g05990	-0.6	At4g13660	-0.6	At5g46280	-0.6	At2g46720	-0.7
At3g06840	-0.6	At4g18640	-0.6	At5g46290	-0.6	At2g28840	-0.7
At3g22380	-0.6	At4g20890	-0.6	At5g46250	-0.6	At2g25730	-0.7
At3g14240	-0.6	At4g21150	-0.6	At5g46840	-0.6	At2g42310	-0.7
At3g15630	-0.6	At4g21470	-0.6	At5g47720	-0.6	At2g32240	-0.7
At3g25860	-0.6	At4g22120	-0.6	At5g49460	-0.6	At2g16640	-0.7
At3g19450	-0.6	At4g22130	-0.6	At5g49980	-0.6	At1g23750	-0.7
At3g20810	-0.6	At4g22570	-0.6	At5g50375	-0.6	At1g52150	-0.7
At3g25560	-0.6	At4g23820	-0.6	At5g52510	-0.6	At1g08600	-0.7
At3g25150	-0.6	At4g24240	-0.6	At5g53140	-0.6	At1g05210	-0.7
At3g27100	-0.6	At4g24620	-0.6	At5g55480	-0.6	At1g10120	-0.7
At3g13290	-0.6	At4g26610	-0.6	At5g56500	-0.6	At1g04110	-0.7
At3g13350	-0.6	At4g31290	-0.6	At5g57410	-0.6	At1g04140	-0.7
At3g25710	-0.6	At4g32460	-0.6	At5g60550	-0.6	At1g04120	-0.7
At3g24240	-0.6	At4g32940	-0.6	At5g61020	-0.6	At1g62020	-0.7
At3g13110	-0.6	At4g33400	-0.6	At5g62190	-0.6	At1g09210	-0.7
At3g23810	-0.6	At4g33530	-0.6	At5g67070	-0.6	At2g36910	-0.7
At3g27300	-0.6	At4g34200	-0.6	At5g04870	-0.6	At2g36880	-0.7
At3g20050	-0.6	At4g34110	-0.6	At5g36290	-0.6	At2g21380	-0.7
At3g20590	-0.6	At4g34390	-0.6	At1g16560	-0.6	At2g01910	-0.7
At3g22880	-0.6	At4g38600	-0.6	At5g20960	-0.6	At2g18160	-0.7
At3g22960	-0.6	At4g39820	-0.6	At5g28290	-0.6	At1g75680	-0.7
At1g66530	-0.6	At3g50370	-0.6	At1g44110	-0.6	At1g43580	-0.7
At1g30410	-0.6	At3g50600	-0.6	At4g17100	-0.6	At1g06620	-0.7
At1g30360	-0.6	At3g55420	-0.6	At4g14010	-0.6	At1g34245	-0.7
At3g12610	-0.6	At3g56480	-0.6	At4g17340	-0.6	At1g71010	-0.7
At3g66658	-0.6	At3g57290	-0.6	At4g15830	-0.6	At1g53910	-0.7
At3g11340	-0.6	At3g57550	-0.6	At4g13940	-0.6	At1g56150	-0.7
At1g30110	-0.6	At3g60300	-0.6	At4g15093	-0.6	At1g35580	-0.7
At1g55130	-0.6	At3g60860	-0.6	At4g15550	-0.6	At1g33811	-0.7
At1g18250	-0.6	At3g60600	-0.6	At2g41630	-0.6	At1g04820	-0.7
At1g07260	-0.6	At5g03870	-0.6	At2g26760	-0.7	At1g01300	-0.7
At2g33570	-0.6	At5g05460	-0.6	At2g30050	-0.7	At1g12160	-0.7
At1g25580	-0.6	At5g06750	-0.6	At2g44120	-0.7	At1g21440	-0.7
At4g00230	-0.6	At5g07960	-0.6	At2g39580	-0.7	At1g49230	-0.7
At4g01120	-0.6	At5g08560	-0.6	At2g34560	-0.7	At1g48950	-0.7
At4g03390	-0.6	At5g11890	-0.6	At2g29980	-0.7	At1g49010	-0.7
At4g03210	-0.6	At5g13390	-0.6	At2g22840	-0.7	At1g19360	-0.7
At4g11830	-0.6	At5g14540	-0.6	At2g02960	-0.7	At1g19330	-0.7
At4g12280	-0.6	At5g19090	-0.6	At2g46930	-0.7	At1g53300	-0.7
At4g12880	-0.6	At5g38140	-0.6	At2g03200	-0.7	At2g43680	-0.7

Supplementary Table S3. Continued.

AGI code	*n*-fold change (log2)	AGI code	*n*-fold change (log2)	AGI code	*n*-fold change (log2)	AGI code	*n*-fold change (log2)
At2g41820	-0.7	At4g10120	-0.7	At5g45700	-0.7	At2g16630	-0.8
At1g47270	-0.7	At4g12390	-0.7	At5g46700	-0.7	At1g62560	-0.8
At1g69810	-0.7	At4g12650	-0.7	At5g47770	-0.7	At1g03850	-0.8
At1g52760	-0.7	At4g12730	-0.7	At5g48250	-0.7	At1g09770	-0.8
At1g71920	-0.7	At4g18970	-0.7	At5g54670	-0.7	At1g09780	-0.8
At1g67920	-0.7	At4g19960	-0.7	At5g55930	-0.7	At2g17790	-0.8
At1g74160	-0.7	At4g20270	-0.7	At5g57610	-0.7	At1g09530	-0.8
At1g29395	-0.7	At4g23800	-0.7	At5g58090	-0.7	At1g12010	-0.8
At1g35260	-0.7	At4g24190	-0.7	At5g60930	-0.7	At1g09280	-0.8
At1g15690	-0.7	At4g29330	-0.7	At5g61340	-0.7	At2g22125	-0.8
At1g02400	-0.7	At4g31340	-0.7	At5g61660	-0.7	At2g38800	-0.8
At3g16470	-0.7	At4g34490	-0.7	At5g61790	-0.7	At2g16060	-0.8
At3g16450	-0.7	At4g35300	-0.7	At5g61840	-0.7	At1g75500	-0.8
At3g01720	-0.7	At4g35380	-0.7	At5g62550	-0.7	At1g20840	-0.8
At3g05420	-0.7	At4g35890	-0.7	At5g65630	-0.7	At1g16370	-0.8
At3g05020	-0.7	At4g37640	-0.7	At5g65700	-0.7	At1g16520	-0.8
At3g01930	-0.7	At4g38430	-0.7	At5g65970	-0.7	At1g75820	-0.8
At3g06300	-0.7	At4g39200	-0.7	At5g66560	-0.7	At1g49310	-0.8
At3g03160	-0.7	At4g39860	-0.7	At5g26360	-0.7	At1g48230	-0.8
At3g03190	-0.7	At4g40060	-0.7	At5g27400	-0.7	At1g52690	-0.8
At3g02910	-0.7	At3g42150	-0.7	At5g14920	-0.7	At1g02840	-0.8
At3g02880	-0.7	At3g44190	-0.7	At5g16250	-0.7	At1g64460	-0.8
At3g22440	-0.7	At3g45010	-0.7	At1g58110	-0.7	At1g80830	-0.8
At3g17680	-0.7	At3g49350	-0.7	At3g51860	-0.7	At1g10640	-0.8
At3g26810	-0.7	At3g52470	-0.7	At4g36670	-0.7	At1g14460	-0.8
At3g15640	-0.7	At3g61990	-0.7	At5g13100	-0.7	At1g07890	-0.8
At3g14010	-0.7	At3g62360	-0.7	At1g44170	-0.7	At1g45145	-0.8
At3g25980	-0.7	At3g62600	-0.7	At1g51060	-0.7	At1g02500	-0.8
At3g16200	-0.7	At5g01220	-0.7	At4g17110	-0.7	At1g53180	-0.8
At3g20390	-0.7	At5g01870	-0.7	At4g17615	-0.7	At1g80280	-0.8
At3g27240	-0.7	At5g03170	-0.7	At4g14400	-0.7	At1g66350	-0.8
At3g23820	-0.7	At5g10180	-0.7	At4g14960	-0.7	At1g67870	-0.8
At3g14840	-0.7	At5g11670	-0.7	At2g23350	-0.7	At1g76460	-0.8
At1g33250	-0.7	At5g13840	-0.7	At2g39900	-0.8	At1g76790	-0.8
At3g12580	-0.7	At5g14690	-0.7	At2g44640	-0.8	At1g71070	-0.8
At1g16920	-0.7	At5g18460	-0.7	At2g46900	-0.8	At1g60960	-0.8
At1g16860	-0.7	At5g23010	-0.7	At2g41310	-0.8	At1g63100	-0.8
At1g07070	-0.7	At5g37600	-0.7	At2g46650	-0.8	At1g28110	-0.8
At1g19870	-0.7	At5g39110	-0.7	At2g18730	-0.8	At3g07660	-0.8
At4g00630	-0.7	At5g40020	-0.7	At2g11260	-0.8	At3g07320	-0.8
At4g01850	-0.7	At5g42630	-0.7	At2g19600	-0.8	At3g08680	-0.8
At4g02200	-0.7	At5g43060	-0.7	At2g37050	-0.8	At3g02790	-0.8

Supplementary Table S3. Continued.

AGI code	*n*-fold change (log2)
At3g14280	-0.8
At3g21550	-0.8
At3g18170	-0.8
At3g23670	-0.8
At3g14680	-0.8
At3g23160	-0.8
At3g12680	-0.8
At3g23890	-0.8
At3g26440	-0.8
At3g10980	-0.8
At3g12110	-0.8
At1g22170	-0.8
At4g02090	-0.8
At4g03205	-0.8
At4g08770	-0.8
At4g13510	-0.8
At4g13770	-0.8
At4g19980	-0.8
At4g20430	-0.8
At4g23480	-0.8
At4g24120	-0.8
At4g26620	-0.8
At4g28260	-0.8
At4g30020	-0.8
At4g35020	-0.8
At4g37740	-0.8
At4g37870	-0.8
At4g39260	-0.8
At4g39900	-0.8
At3g44050	-0.8
At3g46590	-0.8
At3g53190	-0.8
At3g54560	-0.8
At3g58990	-0.8
At3g62980	-0.8
At3g63400	-0.8
At5g01160	-0.8
At5g02100	-0.8
At5g06050	-0.8
At5g08260	-0.8
At5g09760	-0.8
At5g11790	-0.8

AGI code	*n*-fold change (log2)
At5g12100	-0.8
At5g12250	-0.8
At5g18690	-0.8
At5g23530	-0.8
At5g24620	-0.8
At5g42880	-0.8
At5g44480	-0.8
At5g48940	-0.8
At5g49170	-0.8
At5g50200	-0.8
At5g51050	-0.8
At5g55180	-0.8
At5g56040	-0.8
At5g56870	-0.8
At5g59780	-0.8
At5g61380	-0.8
At5g64410	-0.8
At5g65790	-0.8
At5g66920	-0.8
At5g25350	-0.8
At5g27920	-0.8
At5g15580	-0.8
At5g19970	-0.8
At5g28540	-0.8
At4g17215	-0.8
At5g12420	-0.8
At2g39770	-0.8
At2g19110	-0.9
At2g33810	-0.9
At2g40080	-0.9
At2g38370	-0.9
At2g39210	-0.9
At2g35120	-0.9
At2g47630	-0.9
At2g43100	-0.9
At2g38970	-0.9
At2g15120	-0.9
At2g25590	-0.9
At2g01150	-0.9
At1g60740	-0.9
At1g02110	-0.9
At2g42870	-0.9

AGI code	*n*-fold change (log2)
At2g47180	-0.9
At1g05710	-0.9
At1g03060	-0.9
At2g18140	-0.9
At1g59960	-0.9
At1g14890	-0.9
At1g10850	-0.9
At1g28680	-0.9
At1g14210	-0.9
At1g50240	-0.9
At1g78060	-0.9
At1g65860	-0.9
At1g50010	-0.9
At1g49750	-0.9
At1g01780	-0.9
At1g12845	-0.9
At1g04800	-0.9
At1g29160	-0.9
At1g78240	-0.9
At1g49200	-0.9
At1g19540	-0.9
At1g68880	-0.9
At1g13300	-0.9
At3g02230	-0.9
At3g04810	-0.9
At3g10420	-0.9
At3g05730	-0.9
At3g05640	-0.9
At3g03270	-0.9
At3g14350	-0.9
At3g25900	-0.9
At3g21240	-0.9
At3g18710	-0.9
At3g12830	-0.9
At3g19100	-0.9
At3g19130	-0.9
At3g24660	-0.9
At3g24670	-0.9
At3g22120	-0.9
At3g29410	-0.9
At3g52370	-0.9
At1g54970	-0.9

AGI code	*n*-fold change (log2)
At1g48750	-0.9
At1g13670	-0.9
At4g00440	-0.9
At4g00500	-0.9
At4g04770	-0.9
At4g09990	-0.9
At4g19030	-0.9
At4g19120	-0.9
At4g25100	-0.9
At4g27860	-0.9
At4g29380	-0.9
At4g33750	-0.9
At4g40070	-0.9
At3g50240	-0.9
At3g53670	-0.9
At3g54590	-0.9
At3g61130	-0.9
At5g01040	-0.9
At5g01310	-0.9
At5g03350	-0.9
At5g04470	-0.9
At5g05570	-0.9
At5g10430	-0.9
At5g17330	-0.9
At5g22630	-0.9
At5g37740	-0.9
At5g41040	-0.9
At5g43710	-0.9
At5g44030	-0.9
At5g50120	-0.9
At5g51010	-0.9
At5g51600	-0.9
At5g55730	-0.9
At5g57830	-0.9
At5g60690	-0.9
At5g61420	-0.9
At5g62520	-0.9
At5g62680	-0.9
At5g63810	-0.9
At5g65020	-0.9
At5g65210	-0.9
At5g15530	-0.9

Supplementary Table S3. Continued.

AGI code	*n*-fold change (log2)	AGI code	*n*-fold change (log2)	AGI code	*n*-fold change (log2)	AGI code	*n*-fold change (log2)
At5g11090	-0.9	At4g33260	-1.0	At1g61820	-1.1	At4g22010	-1.1
At4g17790	-0.9	At4g35730	-1.0	At2g36290	-1.1	At4g22610	-1.1
At5g12470	-0.9	At3g42950	-1.0	At2g36490	-1.1	At4g24780	-1.1
At2g30500	-1.0	At3g47860	-1.0	At2g36570	-1.1	At4g29080	-1.1
At2g34680	-1.0	At3g53480	-1.0	At2g21540	-1.1	At4g30450	-1.1
At2g44080	-1.0	At3g54400	-1.0	At2g13610	-1.1	At4g36360	-1.1
At2g31010	-1.0	At3g54920	-1.0	At2g01830	-1.1	At3g52720	-1.1
At2g02120	-1.0	At3g55920	-1.0	At2g04160	-1.1	At2g45470	-1.1
At2g17280	-1.0	At3g57040	-1.0	At1g16410	-1.1	At5g10820	-1.1
At2g17820	-1.0	At3g61820	-1.0	At1g14120	-1.1	At5g14230	-1.1
At1g09240	-1.0	At3g62760	-1.0	At1g71830	-1.1	At5g19110	-1.1
At2g36885	-1.0	At3g63240	-1.0	At1g18880	-1.1	At5g23400	-1.1
At2g37040	-1.0	At5g37500	-1.0	At1g20160	-1.1	At5g40390	-1.1
At2g04780	-1.0	At5g38030	-1.0	At1g45130	-1.1	At5g44400	-1.1
At1g75780	-1.0	At5g38150	-1.0	At1g06080	-1.1	At5g44730	-1.1
At1g64140	-1.0	At5g40760	-1.0	At1g73260	-1.1	At5g46230	-1.1
At1g68600	-1.0	At5g42590	-1.0	At1g72700	-1.1	At5g47000	-1.1
At1g11545	-1.0	At5g43180	-1.0	At1g52190	-1.1	At5g48560	-1.1
At1g21100	-1.0	At5g43910	-1.0	At1g77760	-1.1	At5g48570	-1.1
At1g41830	-1.0	At5g44110	-1.0	At1g21250	-1.1	At5g54650	-1.1
At1g52940	-1.0	At5g48070	-1.0	At1g20390	-1.1	At5g57110	-1.1
At1g17430	-1.0	At5g48485	-1.0	At3g16440	-1.1	At5g58150	-1.1
At1g49240	-1.0	At5g48800	-1.0	At3g01290	-1.1	At5g58300	-1.1
At2g41800	-1.0	At5g50760	-1.0	At3g09260	-1.1	At5g25110	-1.1
At1g71410	-1.0	At5g52310	-1.0	At3g10910	-1.1	At3g51895	-1.1
At1g72670	-1.0	At5g60520	-1.0	At3g22790	-1.1	At4g36990	-1.1
At1g77690	-1.0	At5g60530	-1.0	At3g21510	-1.1	At1g26270	-1.1
At1g28010	-1.0	At5g61780	-1.0	At3g19370	-1.1	At2g47500	-1.1
At3g16390	-1.0	At5g63940	-1.0	At3g23170	-1.1	At2g45430	-1.1
At3g16460	-1.0	At5g67430	-1.0	At3g15950	-1.1	At2g41660	-1.1
At3g10340	-1.0	At5g04860	-1.0	At3g12700	-1.1	At2g02130	-1.2
At3g16920	-1.0	At1g31710	-1.0	At3g13310	-1.1	At2g02100	-1.2
At3g12920	-1.0	At5g19790	-1.0	At3g15560	-1.1	At2g37420	-1.2
At3g15030	-1.0	At2g26440	-1.0	At3g27200	-1.1	At2g13820	-1.2
At3g20510	-1.0	At2g30490	-1.1	At3g13650	-1.1	At2g19990	-1.2
At3g22540	-1.0	At2g34910	-1.1	At3g52190	-1.1	At2g37180	-1.2
At3g20015	-1.0	At2g38360	-1.1	At1g54890	-1.1	At2g16660	-1.2
At4g12080	-1.0	At2g02990	-1.1	At1g69526	-1.1	At1g24280	-1.2
At4g12030	-1.0	At2g46140	-1.1	At1g13600	-1.1	At1g79110	-1.2
At4g20110	-1.0	At2g19570	-1.1	At1g07050	-1.1	At2g22500	-1.2
At4g26470	-1.0	At1g24575	-1.1	At4g08390	-1.1	At2g16270	-1.2
At4g27900	-1.0	At1g24260	-1.1	At4g21830	-1.1	At2g31390	-1.2

Supplementary Table S3. Continued.

AGI code	*n*-fold change (log2)	AGI code	*n*-fold change (log2)	AGI code	*n*-fold change (log2)	AGI code	*n*-fold change (log2)
At1g21010	-1.2	At1g31320	-1.2	At5g41920	-1.3	At1g56680	-1.4
At1g02810	-1.2	At1g08430	-1.2	At5g49080	-1.3	At4g17670	-1.4
At1g02950	-1.2	At1g69040	-1.2	At5g57760	-1.3	At2g39850	-1.4
At1g80080	-1.2	At2g39700	-1.3	At5g65640	-1.3	At2g41090	-1.5
At1g11460	-1.2	At2g39980	-1.3	At5g19890	-1.3	At2g39510	-1.5
At1g50060	-1.2	At2g18980	-1.3	At1g67750	-1.3	At2g37130	-1.5
At1g01900	-1.2	At2g14580	-1.3	At2g44380	-1.4	At1g62480	-1.5
At1g33170	-1.2	At2g14560	-1.3	At2g25980	-1.4	At1g67330	-1.5
At1g01640	-1.2	At2g19970	-1.3	At2g28960	-1.4	At1g04680	-1.5
At1g07610	-1.2	At2g01950	-1.3	At2g45050	-1.4	At2g42570	-1.5
At1g79620	-1.2	At1g05000	-1.3	At2g24300	-1.4	At1g30750	-1.5
At1g72200	-1.2	At1g62500	-1.3	At1g23140	-1.4	At1g14730	-1.5
At3g16340	-1.2	At1g60680	-1.3	At1g09750	-1.4	At1g28400	-1.5
At3g24520	-1.2	At2g35860	-1.3	At2g36830	-1.4	At1g69170	-1.5
At3g22600	-1.2	At2g22170	-1.3	At2g28670	-1.4	At1g76800	-1.5
At4g00700	-1.2	At1g06640	-1.3	At1g65840	-1.4	At1g64390	-1.5
At4g13580	-1.2	At1g17190	-1.3	At1g62800	-1.4	At3g23295	-1.5
At4g23690	-1.2	At1g47480	-1.3	At1g47840	-1.4	At3g23190	-1.5
At4g27730	-1.2	At1g30510	-1.3	At1g50110	-1.4	At3g25130	-1.5
At4g31140	-1.2	At1g44970	-1.3	At1g64380	-1.4	At3g20370	-1.5
At4g32690	-1.2	At2g43620	-1.3	At3g21190	-1.4	At3g17420	-1.5
At4g34600	-1.2	At1g11080	-1.3	At3g23150	-1.4	At3g13790	-1.5
At4g35060	-1.2	At1g70710	-1.3	At3g23290	-1.4	At4g04955	-1.5
At4g35150	-1.2	At3g01670	-1.3	At3g26470	-1.4	At4g05200	-1.5
At3g50300	-1.2	At3g09940	-1.3	At2g40530	-1.4	At4g11190	-1.5
At3g50640	-1.2	At3g07210	-1.3	At1g31950	-1.4	At4g24110	-1.5
At5g01890	-1.2	At3g22231	-1.3	At4g01480	-1.4	At4g24890	-1.5
At5g02540	-1.2	At1g17860	-1.3	At4g24140	-1.4	At3g54770	-1.5
At5g06800	-1.2	At4g12400	-1.3	At4g30190	-1.4	At3g54960	-1.5
At5g13870	-1.2	At4g19810	-1.3	At4g30320	-1.4	At5g37990	-1.5
At5g22740	-1.2	At4g30140	-1.3	At3g47420	-1.4	At5g39320	-1.5
At5g44380	-1.2	At4g32870	-1.3	At3g53620	-1.4	At5g48010	-1.5
At5g51060	-1.2	At3g44400	-1.3	At3g56040	-1.4	At5g62165	-1.5
At5g63650	-1.2	At3g44540	-1.3	At3g56400	-1.4	At5g63560	-1.5
At5g66690	-1.2	At3g48100	-1.3	At5g01210	-1.4	At1g51840	-1.5
At5g67210	-1.2	At3g62270	-1.3	At5g40510	-1.4	At5g20410	-1.5
At5g15740	-1.2	At5g03670	-1.3	At5g42180	-1.4	At5g20830	-1.5
At1g31850	-1.2	At5g05340	-1.3	At5g48290	-1.4	At1g62660	-1.6
At4g37010	-1.2	At5g07030	-1.3	At5g64570	-1.4	At2g36100	-1.6
At5g20550	-1.2	At5g14150	-1.3	At5g25890	-1.4	At1g10480	-1.6
At5g20630	-1.2	At5g15230	-1.3	At5g27930	-1.4	At1g15210	-1.6
At5g09220	-1.2	At5g23240	-1.3	At5g20740	-1.4	At1g74210	-1.6

Supplementary Table S3. Continued.

AGI code	*n*-fold change (log2)
At3g06035	-1.6
At3g26200	-1.6
At3g13175	-1.6
At1g66880	-1.6
At4g29270	-1.6
At4g30110	-1.6
At3g51710	-1.6
At3g59900	-1.6
At3g63200	-1.6
At5g05500	-1.6
At5g05790	-1.6
At5g12940	-1.6
At5g22500	-1.6
At5g53250	-1.6
At5g57240	-1.6
At5g58860	-1.6
At5g66280	-1.6
At1g77330	-1.6
At1g51830	-1.6
At2g38080	-1.7
At2g03020	-1.7
At2g41230	-1.7
At2g15390	-1.7
At2g04025	-1.7
At1g31350	-1.7
At1g68650	-1.7
At1g22440	-1.7
At1g08310	-1.7
At1g29280	-1.7
At1g21310	-1.7
At3g22620	-1.7
At3g22240	-1.7
At3g19550	-1.7
At4g18610	-1.7
At3g49860	-1.7
At5g09520	-1.7
At5g62340	-1.7
At5g63600	-1.7
At5g19520	-1.7
At4g16260	-1.7
At2g34390	-1.8
At2g47880	-1.8

AGI code	*n*-fold change (log2)
At1g08650	-1.8
At2g01890	-1.8
At1g64590	-1.8
At1g29050	-1.8
At1g72360	-1.8
At1g68850	-1.8
At3g05890	-1.8
At3g17790	-1.8
At3g19390	-1.8
At3g27220	-1.8
At1g67030	-1.8
At4g21600	-1.8
At4g29690	-1.8
At3g43670	-1.8
At5g15120	-1.8
At5g22580	-1.8
At5g55450	-1.8
At5g66170	-1.8
At4g37070	-1.8
At4g15210	-1.8
At3g29970	-1.8
At2g25810	-1.9
At2g04460	-1.9
At2g01880	-1.9
At1g11670	-1.9
At1g62770	-1.9
At1g49210	-1.9
At1g66280	-1.9
At1g67860	-1.9
At1g52240	-1.9
At1g64360	-1.9
At3g21770	-1.9
At1g16850	-1.9
At1g22030	-1.9
At4g27450	-1.9
At4g28250	-1.9
At4g35350	-1.9
At3g52430	-1.9
At5g17820	-1.9
At5g22890	-1.9
At5g37690	-1.9
At5g43780	-1.9

AGI code	*n*-fold change (log2)
At5g59520	-1.9
At5g64100	-1.9
At4g17800	-1.9
At2g37750	-2.0
At2g38390	-2.0
At2g46740	-2.0
At2g18370	-2.0
At1g61590	-2.0
At1g05260	-2.0
At1g12110	-2.0
At1g64780	-2.0
At1g15100	-2.0
At1g01750	-2.0
At4g31320	-2.0
At4g31730	-2.0
At5g20150	-2.0
At1g28290	-2.0
At2g31030	-2.1
At2g38940	-2.1
At2g36090	-2.1
At1g76650	-2.1
At1g49860	-2.1
At1g18980	-2.1
At3g01190	-2.1
At3g10040	-2.1
At3g25790	-2.1
At3g49160	-2.1
At5g23020	-2.1
At5g39890	-2.1
At5g44550	-2.1
At2g35380	-2.2
At2g18210	-2.2
At1g77120	-2.2
At1g22990	-2.2
At2g21100	-2.2
At2g47200	-2.2
At1g74500	-2.2
At3g03790	-2.2
At3g02550	-2.2
At3g15240	-2.2
At4g13290	-2.2
At4g22212	-2.2

AGI code	*n*-fold change (log2)
At5g09530	-2.2
At5g10580	-2.2
At5g48000	-2.2
At5g26280	-2.2
At2g22860	-2.3
At2g29330	-2.3
At1g05250	-2.3
At1g12080	-2.3
At2g04800	-2.3
At2g17080	-2.3
At1g73120	-2.3
At3g01260	-2.3
At3g02040	-2.3
At3g23090	-2.3
At3g19710	-2.3
At4g02290	-2.3
At4g18780	-2.3
At4g25090	-2.3
At4g26320	-2.3
At4g33070	-2.3
At3g57950	-2.3
At5g05960	-2.3
At5g38710	-2.3
At5g40730	-2.3
At5g60950	-2.3
At5g09480	-2.3
At2g19590	-2.4
At2g17850	-2.4
At2g48130	-2.4
At2g48140	-2.4
At1g11580	-2.4
At3g23430	-2.4
At3g14530	-2.4
At3g25190	-2.4
At3g06140	-2.4
At4g29140	-2.4
At3g58550	-2.4
At5g17340	-2.4
At5g43370	-2.4
At5g56540	-2.4
At5g63660	-2.4
At2g32990	-2.5

Supplementary Table S3. Continued.

AGI code	*n*-fold change (log2)
At2g44790	-2.5
At2g40300	-2.5
At1g14960	-2.5
At3g14940	-2.5
At4g20390	-2.5
At5g59090	-2.5
At2g30210	-2.6
At1g31050	-2.6
At1g02900	-2.6
At2g43535	-2.6
At1g74460	-2.6
At3g45710	-2.6
At5g04960	-2.6
At5g51720	-2.6
At2g44010	-2.7
At2g21045	-2.7
At2g11810	-2.7
At2g43870	-2.7
At3g04320	-2.7
At4g02270	-2.7
At4g12510	-2.7
At4g29800	-2.7
At4g40090	-2.7
At3g54260	-2.7
At3g56090	-2.7
At5g10040	-2.7
At5g23830	-2.7
At5g47740	-2.7
At5g47990	-2.7
At5g15600	-2.7
At4g14980	-2.7
At2g16750	-2.8
At1g09090	-2.8
At2g36120	-2.8
At1g33750	-2.8
At1g69230	-2.8
At1g74310	-2.8
At3g03540	-2.8
At4g25760	-2.8
At3g60490	-2.8
At5g26260	-2.8
At1g73330	-2.8
At2g33790	-2.9
At2g40230	-2.9
At1g54540	-2.9
At1g15045	-2.9
At4g00890	-2.9
At4g13280	-2.9
At4g30670	-2.9
At5g54370	-2.9
At5g66390	-2.9
At2g46880	-3.0
At2g42610	-3.0
At1g33055	-3.0
At1g75040	-3.0
At1g13420	-3.0
At3g25930	-3.0
At3g20340	-3.0
At2g40480	-3.0
At3g45160	-3.0
At5g57530	-3.0
At4g15340	-3.0
At2g45220	-3.0
At2g28780	-3.1
At2g18660	-3.1
At3g13610	-3.1
At3g12500	-3.1
At4g10270	-3.1
At4g11310	-3.1
At5g16560	-3.1
At5g44610	-3.1
At5g46900	-3.1
At4g14130	-3.1
At2g22920	-3.2
At1g78815	-3.2
At3g25830	-3.2
At3g44990	-3.2
At3g54040	-3.2
At3g62040	-3.2
At5g57090	-3.2
At4g14630	-3.2
At2g47270	-3.3
At1g72230	-3.3
At3g55720	-3.3
At5g10130	-3.3
At2g23540	-3.4
At2g42380	-3.4
At1g52060	-3.4
At2g43590	-3.4
At1g02360	-3.4
At4g26010	-3.4
At4g30170	-3.4
At4g33560	-3.4
At3g43190	-3.4
At5g25160	-3.4
At2g29750	-3.5
At2g43610	-3.5
At3g05630	-3.5
At4g38080	-3.5
At1g07880	-3.6
At3g15230	-3.6
At4g25820	-3.6
At3g44520	-3.6
At3g61410	-3.6
At2g23050	-3.6
At1g80240	-3.7
At3g24290	-3.7
At3g29780	-3.7
At1g19200	-3.7
At2g05510	-3.8
At1g30870	-3.8
At3g16670	-3.8
At4g26220	-3.8
At3g57010	-3.8
At5g01600	-3.8
At1g05650	-3.9
At1g67865	-3.9
At3g55670	-3.9
At3g62680	-3.9
At2g45130	-4.0
At1g31060	-4.0
At1g73010	-4.0
At1g17710	-4.0
At5g60760	-4.0
At1g51280	-4.1
At1g64920	-4.1
At1g34510	-4.1
At2g30340	-4.1
At4g19430	-4.1
At4g37410	-4.2
At2g01530	-4.3
At1g70850	-4.3
At1g73220	-4.3
At1g12740	-4.3
At5g23840	-4.3
At5g27480	-4.3
At1g32450	-4.4
At3g06390	-4.4
At3g59730	-4.4
At5g10210	-4.4
At5g20790	-4.4
At4g14060	-4.4
At2g21650	-4.5
At4g12550	-4.5
At3g51330	-4.5
At5g15070	-4.5
At1g19530	-4.6
At4g34580	-4.7
At1g78990	-4.7
At1g52070	-4.8
At4g15390	-4.8
At2g14210	-4.9
At5g35940	-4.9
At2g27000	-5.0
At3g13760	-5.0
At4g00680	-5.0
At3g49960	-5.0
At1g14080	-5.1
At4g00080	-5.1
At5g66985	-5.1
At1g73300	-5.1
At1g43800	-5.2
At5g35190	-5.6
At5g67400	-6.1
At4g39675	-7.5
At2g01520	-9.8
